# A comparison of epigenetic mitotic-like clocks for cancer risk prediction

**DOI:** 10.1186/s13073-020-00752-3

**Published:** 2020-06-24

**Authors:** Andrew E. Teschendorff

**Affiliations:** 1 CAS Key Laboratory of Computational Biology, CAS-MPG Partner Institute for Computational Biology, Shanghai Institute of Nutrition and Health, Shanghai Institute for Biological Sciences, University of Chinese Academy of Sciences, Chinese Academy of Sciences, 320 Yue Yang Road, Shanghai, 200031 China; 2 UCL Cancer Institute, University College London, 72 Huntley Street, London, WC1E 6BT UK

**Keywords:** DNA methylation, Epigenetic clock, Cancer, Mitotic, Stem cells, Aging

## Abstract

**Background:**

DNA methylation changes that accrue in the stem cell pool of an adult tissue in line with the cumulative number of cell divisions may contribute to the observed variation in cancer risk among tissues and individuals. Thus, the construction of epigenetic “mitotic” clocks that can measure the lifetime number of stem cell divisions is of paramount interest.

**Methods:**

Building upon a dynamic model of DNA methylation gain in unmethylated CpG-rich regions, we here derive a novel mitotic clock (“epiTOC2”) that can directly estimate the cumulative number of stem cell divisions in a tissue. We compare epiTOC2 to a different mitotic model, based on hypomethylation at solo-WCGW sites (“HypoClock”), in terms of their ability to measure mitotic age of normal adult tissues and predict cancer risk.

**Results:**

Using epiTOC2, we estimate the intrinsic stem cell division rate for different normal tissue types, demonstrating excellent agreement (Pearson correlation = 0.92, *R*^2^ = 0.85, *P* = 3e−6) with those derived from experiment. In contrast, HypoClock’s estimates do not (Pearson correlation = 0.30, *R*^2^ = 0.09, *P* = 0.29). We validate these results in independent datasets profiling normal adult tissue types. While both epiTOC2 and HypoClock correctly predict an increased mitotic rate in cancer, epiTOC2 is more robust and significantly better at discriminating preneoplastic lesions characterized by chronic inflammation, a major driver of tissue turnover and cancer risk. Our data suggest that DNA methylation loss at solo-WCGWs is significant only when cells are under high replicative stress and that epiTOC2 is a better mitotic age and cancer risk prediction model for normal adult tissues.

**Conclusions:**

These results have profound implications for our understanding of epigenetic clocks and for developing cancer risk prediction or early detection assays. We propose that measurement of DNAm at the 163 epiTOC2 CpGs in adult pre-neoplastic lesions, and potentially in serum cell-free DNA, could provide the basis for building feasible pre-diagnostic or cancer risk assays. epiTOC2 is freely available from 10.5281/zenodo.2632938

## Background

There is increasing evidence that the risk of neoplastic transformation of any given tissue in any given individual is a direct function of the mitotic age of the tissue, that is, cancer risk may correlate with the cumulative number of cell divisions within the underlying (adult) stem cell pool [1–4]. The mitotic age of a tissue depends on intrinsic factors, such as the cell turnover rate of the tissue, and on tissue-independent factors that modulate this normal turnover rate. Well-known factors that increase the turnover rate of a tissue are chronic inflammation and tissue injury/repair [5], which may result from exogenous cancer risk factors such as bacterial/viral infections or smoke carcinogens [6–8]. Increased cellular turnover in a tissue is thought to underpin the gradual accumulation of molecular alterations in the stem cell pool, eventually predisposing specific subclones to neoplastic transformation [9, 10].

Given the appeal and importance of such a mitotic stem cell model of oncogenesis, there is increased interest to construct molecular “mitotic-like” clocks that can yield proxies for the cumulative number of stem cell divisions in the tissue of any given individual, which may ultimately serve to predict the risk of neoplastic transformation [11–21]. DNA methylation (DNAm)-based mitotic-like clocks [15–17], which aim to track the cumulative number of DNA methylation errors arising during cell division [13, 14, 22], are of particular interest given that DNAm changes in normal tissue have already been shown to correlate with cancer risk [7, 18, 20, 23–26]. However, two outstanding questions have emerged with regards to these DNAm-based mitotic-like clocks. First, can existing models be used to directly estimate the cumulative number of stem cell divisions in a tissue. While some recent studies have proposed DNAm drift models that can compute the time of onset for premalignant fields (e.g., Barrett’s esophagus) and colorectal neoplasia lesions [27–29], these models have not been used to explicitly calculate stem cell division rates in normal tissues. To achieve this, requires formulation of an explicit mathematical dynamic model for DNAm transmission between cell generations, as well as subsequent derivation of a proxy that describes DNAm measurements in a sample in terms of the underlying number of stem cell divisions. A second outstanding question is which CpGs are best for tracking mitotic age, as two complementary approaches have emerged. One proposal, which underlies the epiTOC model [15], is based on CpG sites in CpG-rich regions marked by the polycomb repressive complex-2 (PRC2) which are generally unmethylated across many different fetal tissue types. The rationale for focusing on these sites is fourfold: they become methylated during ontogeny and aging [13, 15, 22, 30, 31], they are strongly enriched among sites undergoing hypermethylation with age [32–34] and exposure to cancer risk factors [18, 35–37], and fourthly, most of the observed hypermethylation is not functional as it preferentially occurs at target genes that are not expressed in the fetal tissue [38]. However, the underlying molecular mechanism which leads to erroneous methylation accrual at otherwise unmethylated sites is still unclear [39]. An alternative model, recently advocated by Berman and colleagues [17], is focused on “solo-WCGWs,” i.e., isolated CpGs occurring in a WCGW sequence context, which are generally methylated in fetal tissue and which would gradually lose methylation as a result of incomplete methylation maintenance during cell division. Approximately 3.7 million solo-WCGWs were identified, with 1.8 million of these mapping to partially methylated domains (PMDs), which largely overlap with late-replicating regions [40]. While hypomethylation at solo-WCGWs is largely seen in cancer and early development (i.e., states of high replicative stress), it is unclear whether DNAm loss in late-replicating regions would play a sufficiently major role in a normal physiological setting or in pre-cancerous states where cells are not under significant replicative stress. Moreover, solo-WCGWs may be subject to substantial confounding by cell type heterogeneity [41, 42], which may preclude a direct interpretation in terms of DNAm changes that accrue because of cell division.

Here, we address these outstanding questions. We first derive and validate a novel mitotic clock model called epiTOC2 (Epigenetic Timer of Cancer-2), which, unlike our previous epiTOC model [15], allows direct estimation of the cumulative number of stem cell divisions in a tissue. Like epiTOC, epiTOC2 is also based on cumulative hypermethylation at a subset of PRC2 targets. Subsequently, we provide a detailed comparison of epiTOC2 to an analogous model based on cumulative hypomethylation at solo-WCGWs (called “HypoClock”). This comparison is performed in normal adult tissue, precancerous lesions, and cancer itself, confirming our hypothesis that methylation loss at solo-WCGWs is only significant for cells under high replicative stress. In addition, we demonstrate that unlike the CpGs making up epiTOC2, solo-WCGWs are subject to substantial confounding by cell type heterogeneity, which may preclude their use for estimating mitotic age. Overall, our data suggest that epiTOC2 is much better suited than HypoClock for developing cancer risk prediction assays.

## Methods

### Formulation and derivation of epiTOC2

epiTOC2 is based on a formal dynamic model relating DNA methylation, as measured in a sample tissue, to the underlying total number of stem cell divisions per stem cell. It builds upon a site-specific (i.e., CpG specific) model for DNA methylation transmission between cell generations, first proposed by Generaux [43]. To describe this model, we first introduce variables representing the frequency of methylated and unmethylated gametes in the sample at a given instant in time (corresponding to a particular number of cell divisions), as well as the corresponding frequencies of gamete-pairs, which we refer to as dyads:

$$ {\displaystyle \begin{array}{l}{m}_t=\mathrm{frequency}\ \mathrm{of}\ \mathrm{methylated}\ \mathrm{gametes}\ \mathrm{at}\ \mathrm{division}\ \mathrm{time}\ t\\ {}{u}_t=\mathrm{frequency}\ \mathrm{of}\ \mathrm{unmethylated}\ \mathrm{gametes}=1-{m}_t\\ {}{M}_t=\mathrm{frequency}\ \mathrm{of}\ \mathrm{methylated}\ \mathrm{dyads}\ \mathrm{at}\ \mathrm{division}\ \mathrm{time}\ t\\ {}{H}_t=\mathrm{frequency}\ \mathrm{of}\ \mathrm{hemimethylated}\ \mathrm{dyads}\ \mathrm{at}\ \mathrm{division}\ \mathrm{time}\ t\\ {}{U}_t=\mathrm{frequency}\ \mathrm{of}\ \mathrm{unmethylated}\ \mathrm{dyads}=1-{M}_t-{H}_t\end{array}} $$

We also introduce a number of parameters describing the probabilities of methylation maintenance, *μ*, and of de novo methylation on parent *δ*_*p*_ and daughter *δ*_*d*_ strands. Thus, 1-*μ* is the probability of methylation loss of a methylated strand after cell division. We stress that all the introduced parameters are site (i.e., CpG) specific, but for now and for notational convenience, we do not indicate this explicitly. The frequency of fully methylated and hemimethylated dyads at division time *t* can then be expressed as:

$$ {\displaystyle \begin{array}{l}{M}_t=\mu {m}_{t-1}+{\delta}_p{\delta}_d{u}_{t-1}\\ {}{H}_t=\left(1-\mu \right){m}_{t-1}+{\delta}_d\left(1-{\delta}_p\right){u}_{t-1}+{\delta}_p\left(1-{\delta}_d\right){u}_{t-1}\end{array}} $$

Since at any given division time *t*, ${m}_t={M}_t+\frac{1}{2}{H}_t$, *m*_*t*_ can be expressed as

$$ {m}_t=a+b{m}_{t-1} $$

with

$$ {\displaystyle \begin{array}{l}a=\frac{1\ }{2}\left({\delta}_p+{\delta}_d\right)\equiv \frac{1\ }{2}\delta \\ {}b=\frac{1}{2}\left(1+\mu -\delta \right)\end{array}} $$

Importantly, the above iteration equation can be solved exactly, yielding the formula

$$ {m}_t=a\sum \limits_{k=0}^{t-1}{b}^k+{m}_0{b}^t $$

where *m*_*0*_ is the number of methylated gametes at time 0. We shall assume that this time refers to the fetal stage. Using the Taylor expansion ${\left(1-b\right)}^{-1}={\sum}_{k=0}^{\infty }{b}^k$, one can express the above equation as

$$ {m}_t=\frac{a}{1-b}+{b}^t\left({m}_0-\frac{a}{1-b}\right) $$

DNA methylation is normally measured and quantified as a *β* value, representing the fraction of methylated CpGs, and equals

$$ {\beta}_t\equiv {m}_t=\frac{\delta }{2\left(1-b\right)}+{b}^t\left({\beta}_0-\frac{\delta }{2\left(1-b\right)}\right) $$

Next, we derive a useful approximation that allows direct estimation of division rates from the measured DNA methylation profile. First, we note that the probability of methylation loss is typically very small. A number of studies [43, 44] have estimated the probability of methylation maintenance to be approximately 0.95, yet this estimate was derived from analyzing DNAm patterns at only 2 genomic loci. Moreover, methylation maintenance is likely to be CpG dependent. It is therefore unclear if existing estimates can be applied genome-wide. Because we shall focus on sites that start out unmethylated in fetal tissue, it is reasonable, in a 1st approximation, to assume that methylation maintenance is much closer to 1, i.e., *μ* ≈ *1*. It follows that *b ≈ 1 – δ/2*, so that the above equation becomes

$$ {\beta}_t=1-{\left(1-\frac{\delta }{2}\right)}^t+{\beta}_0{\left(1-\frac{\delta }{2}\right)}^t $$

which expresses the DNAm beta value at cell division time *t* in terms of the total de novo methylation probability *δ*, the number of stem cell divisions *t*, and the methylation value at time 0 (*β*_*0*_). Note that, as required, $\underset{t\to \infty }{\lim }{\beta}_t=1$.

The above equation forms the basis for the epiTOC2 model. Assume that DNA methylation has been measured in a sample *s* representing tissue type *x* and that *i* labels a specific CpG site unmethylated in fetal tissues (i.e., *β*_*i0*_ < 0.1), for which its methylation frequency increases according to the above formula, i.e.,

$$ {\beta}_{is(t)}=1-{\left(1-\frac{\delta_i}{2}\right)}^{t\left(s,x\right)}+{\beta}_{i0}{\left(1-\frac{\delta_i}{2}\right)}^{t\left(s,x\right)} $$

where the number of stem cell divisions *t* is obviously dependent on the individual *s* and tissue type *x*. Writing *t*(*s*,*x*) *= TNSC*(*s*,*x*), where *TNSC* stands for the total number of stem cell divisions per stem cell, this number can be factorized as

$$ TNSC\left(s,x\right)=A(s)R\left(s,x\right) $$

where *A*(*s*) denotes the chronological age of individual *s* and where *R*(*s*,*x*) denotes the annual stem cell division rate per stem cell, i.e., the number of divisions a stem cell undergoes during 1 year in tissue *x*. We note that in general, *R*(*s*,*x*) will have two contributions: an intrinsic rate (*IR*) contribution and an extrinsic modulating factor *ER*, so that *R*(*s*,*x*) *= IR*(*x*)(*1 + ER*(*s*,*x*)). It is reasonable that the intrinsic rate is only dependent on the tissue type, but that the modulating extrinsic rate factor is sample dependent, which may include the effects of environmental exposures, as well as inherited genetic factors (which may also modulate the *IR*).

If we have a cohort of reasonably healthy individuals, then we can assume that on average, *R*(*s*,*x*) *= IR*(*x*), and therefore that *TNSC*(*s*,*x*) *= A*(*s*)*IR*(*x*). Thus, by fitting the formula

$$ {\beta}_{is(t)}=1-{\left(1-\frac{\delta_i}{2}\right)}^{A(s) IR(x)}+{\beta}_{i0}{\left(1-\frac{\delta_i}{2}\right)}^{A(s) IR(x)} $$

to a DNAm dataset from a large cohort of healthy individuals of known ages in a single tissue type *x*, and across a sufficiently large number of CpG sites *i*, we can solve for the unknown parameters (*δ*_*i*_, *β*_*i0*,_*IR*(*x*)). Note that the de novo methylation probabilities as well as the ground state methylation values are CpG dependent, but that the intrinsic rate of cell division is not. At a fixed site *i*, variation in the measured beta values across individuals is therefore due to their different ages and due to specific stochastic factors (e.g., exposures), which in a healthy cohort we assume average out to zero.

### CpG selection and estimation of epiTOC2 model parameters

The mathematical approximations derived above assumed CpG sites where DNAm gradually increases with mitotic age. As with our previous epiTOC-model [15], we therefore begin with a set of 385 CpGs that locate to gene promoters marked by the PRC2 complex, which are constitutively unmethylated in a ground state of age zero (e.g., fetal tissue), and which acquire increases in DNAm during hematopoietic ontogeny [31] and aging [15]. Specifically, the 385 epiTOC CpGs were identified as being unmethylated across 37 fetal samples from the Stem-Cell Matrix Compendium-2 (SCM2) [45] and 15 cord blood samples [46].

To estimate the parameters of the epiTOC2 model, a large number of individuals are required. We use whole blood, since for this tissue type DNAm data for large healthy cohorts is available. Specifically, we used the Illumina Infinium 450k data from Hannum et al. [47], encompassing 656 whole blood samples. Model fitting and parameter estimation is performed for each CpG site *i* separately, using non-linear least squares (*nls2* R-package), using a three-dimensional grid of starting values: *δ* = (0.001, 0.00075, 0.0005, 0.00025, 0.0001, 5e−5, 1e−5), *β*_*0*_ = (*0*, *0*.*01*, *0*.*02*, *0*.*03*, *0*.*04*, *0*.*05*), *IR =* (*1*, *2*, *3*, *4*, *5*, *10*, *15*, *20*, *…*, *90*, *95*, *100*). Next, we inspected the individual fits for all 385 epiTOC sites and observed that for some CpGs, the estimated rate of increase in DNAm over a 10-year period (i.e., the product δ*IR*10) was less than 1%. Given that the resolution of the Illumina assay is about 1% [48], we thus decided to only retain CpGs where the product *δ* *IR is larger or equal than 0.001, i.e., if δ*IR*10 is larger or equal than 0.01. This resulted in 163 CpGs (the “epiTOC2” CpGs). However, we note that the true *IR* value cannot be CpG dependent, and therefore, any differences in the estimated *IR* value across these 163 CpGs reflect differences in the de novo methylation probabilities, which however are not optimally captured by the estimation procedure. Thus, to arrive at a single *IR* estimate for blood, we posited that the specific combination (δ, *IR*) of values with highest representation among all 163 CpGs would correspond to the most likely true *IR* value. Remarkably, we observed a clear mode at *δ = 5e*−*5* and *IR = 35*. This *IR* estimate corresponds to a turnover rate of 365/35 ≈ 10, i.e., to blood turnover every 10 days. Finally, we use this *IR* estimate to refit the model, so as to obtain fits for each of the 163 CpGs in terms of a site-specific de novo methylation probability *δ*_*i*_ and ground-state methylation value *β*_*i0*_, all of which are assumed to be independent of tissue type.

### Estimation of mitotic age and relation to previous epiTOC model

We can now estimate the mitotic age, i.e., the total number of stem cell division per stem cell (*TNSC*) of any sample *s*, irrespective of tissue type *x*, using the estimated parameters *δ*_*i*_ and *β*_*i0*._ We note that it is reasonable to assume in a first approximation that these parameter estimates are independent of tissue type, since de novo methylation probabilities are likely to be mostly sequence dependent, whilst the ground-state methylation values are all close to 0 for all fetal tissue types, by virtue of how the original 385 epiTOC CpGs were selected. From the previous formula, we can now derive the first-order approximation

$$ {\displaystyle \begin{array}{l}{\beta}_{is}=1-\left(1-\frac{\delta_i}{2}A(s)R\left(x,s\right)\right)+\left(1-\frac{\delta_i}{2}A(s)R\left(x,s\right)\right){\beta}_{i0}\\ {}=>\kern0.5em {\beta}_{is}={\beta}_{i0}+\frac{\delta_i}{2}\left(1-{\beta}_{i0}\right) TNSC(s)\end{array}} $$

where *A*(*s*) is the chronological age of sample *s*. Importantly, this equation can now be solved directly for *TNSC*(*s*):

$$ TNSC(s)=\frac{2}{n}\sum \limits_{i=1}^n\frac{\beta_{is}-{\beta}_{i0}}{\delta_i\left(1-{\beta}_{i0}\right)} $$

where *n* is the number of CpGs used and equals 163, since this is the number of epiTOC CpGs for which we obtained reliable *δ*_*i*_ and *β*_*i0*_ parameter estimates.

Of note, if we make the further approximation that *β*_*i*0_ ≈ 0 for all *i* (reasonable since all *β*_*i*0_ ≤ 0.05), *TNSC*(*s*) becomes a weighted average of the DNAm beta values over the 163 CpGs:

$$ TNSC(s)=\frac{1}{n}\sum \limits_{i=1}^n{w}_i{\beta}_{is}=\frac{1}{n}\sum \limits_{i=1}^n\frac{2\ {\beta}_{is}}{\delta_i} $$

And if we further assume that the de novo methylation probabilities are site independent, then *TNSC*(*s*) is directly proportional to our previous epiTOC-score [15], denoted by *pcgtAge*, given by the average methylation beta value over selected CpG sites, albeit over the reduced subset of 163 epiTOC2 CpGs (*pcgtAge* was defined as the average DNAm over the 385 epiTOC CpGs). Thus, modulo the specific set of CpG-sites, epiTOC represents a special case of epiTOC2, with epiTOC2 providing a more general assumption-free model that subsumes epiTOC.

### Estimation of the intrinsic stem cell division rate of normal tissues

From the previous formula, we can estimate *TNSC*(*s*) for each sample *s*. If the ages of the samples are known, and assuming that all samples represent the same tissue type *x*, we can now estimate the intrinsic stem cell division rate of a tissue by

$$ IR(x)={\left\langle TNSC\left(s,x\right)/A(s)\right\rangle}_s $$

where <*>_s_ denotes the average or median over the samples. Results reported in this work are for the median estimator, as it is more robust. However, we point out that results are largely unchanged had we used the mean. We used the above formula to estimate the intrinsic rate (*IR*) of stem cell division for normal tissue types, for which genome-wide DNAm data generated in large numbers of samples is available. In one analysis, we used the Illumina 450k DNAm data for the normal adjacent tissue samples of the TCGA [49]. In the second analysis, we obtained and normalized Illumina 450k DNAm data for normal tissues not adjacent to cancer, as described further below.

### Simulation model to demonstrate identifiability of model parameters

To demonstrate that the parameters *IR*, *δ*_*i*_ and *β*_*i0*_ are all identifiable from the non-linear least squares fitting procedure, we simulated age-associated DNAm accrual at 163 loci in a cohort of 656 individuals with an age-distribution identical to that of Hannum et al [47]. Age range was 19 to 101. All samples are from the same tissue, and so *IR* is the same for all samples. In the simulation, we set IR = 35. We also set all *β*_*i0*_ *= 0*.*05*. We choose variable *δ*_*i*_ drawn from values (0.001, 0.00075, 0.0005, 0.00025, 0.0001, 5e−5, 1e−5). To model noise and, specifically, to capture the age-associated increase in DNAm variance, as observed in real data, we used the Hannum et al. dataset to estimate the variance in DNAm for the 385 epiTOC PRC2 loci for samples in specific age intervals. We used the following age intervals: age < 40 (*n* = 35), 40 ≤ age < 50 (*n* = 74), 50 ≤ age < 60 (*n* = 138), 60 ≤ age < 70 (*n* = 167), 70 ≤ age < 80 (*n* = 142), and age ≥ 80 (*n* = 100), yielding sufficient samples in each interval to estimate DNAm variance for each PRC2 locus. We then averaged over the 385 loci to obtain one final DNAm variance estimate for each age interval. To incorporate this variance into the simulation model, we generated a beta value for each of the 163 loci *i* in each sample *s*, by drawing it from a beta-distribution *B*(*a*,*b*) with a mean value given by the formula

$$ \mu =1-{\left(1-\frac{\delta_i}{2}\right)}^{\mathrm{Age}(s) IR}+{\beta}_{i0}{\left(1-\frac{\delta_i}{2}\right)}^{\mathrm{Age}(s) IR} $$

and a variance σ^2^ given by the estimate for the corresponding age interval. It can be shown that the (*a*,*b*) parameters are given by *a =* (*μ*(*1* − *μ*)*/σ*^*2*^ − *1*)*μ* and *b = a/μ* − *a*. Finally, having simulated the DNAm data for the 163 loci and 656 samples, we then fit the original epiTOC2 model as described earlier and compare parameter estimates to the true values.

### Independent estimates of stem cell division rates in normal tissues

Experimentally derived stem cell division rate estimates were obtained from independent studies: from Tomasetti and Vogelstein [1, 50], we obtained estimates for colon (*IR* = 73 divisions per stem cell per year), rectum (*IR* = 73), esophagus (*IR* = 33.2), head and neck (*IR* = 21.5), liver (*IR* = 0.91), lung (*IR* = 0.07), pancreas (*IR* = 1), thyroid (*IR* = 0.087), breast (*IR* = 4.3), prostate (*IR* = 2.99), and kidney (*IR =* 0.91). The turnover rate in gastric stomach tissue has been estimated to be on average about every 5.5 days [51–53], which leads to an *IR* estimate of about 66.4. In the case of bladder, we obtained a value of *IR* = 2 from [54].

Skin is a tissue where the turnover rate is highly dependent on age, with cell renewal occurring every 3 weeks (21 days) for a teenager, every 35 days for an adult between ages 20 and 50, and with cell-turnover increasing from 45 to 90 days as age increases from 50 to 80. Thus, we estimated an average lifetime *IR* for a person of a given age by integration of these estimates, using a constant rate until age 20, another constant rate between the ages of 20 to 50, and finally a linear model for the turnover rate between the ages of 50 and 80. For instance, for a teenager, the lifetime *IR* value is simply 365/21. For a person between the ages of 20 and 50, the lifetime *IR* value is (20*(365/21) + (*age* − 20)*365/35)/*age*, whereas for a person over the age of 50, the lifetime *IR* value was estimated as (20*365/21 + 30*365/35 + (*age* − 50)*365/*rd*)/*age*, where *rd =* (*45/30*)*age + 90–8*15 is the rate of turnover for someone over the age of 50. Thus, for someone of age 50, *rd* = 45, and for age 80, *rd* = 90. Assuming a cohort of mean age 50, the mean lifetime *IR* over the cohort is approximately 13.3. Hence, for skin, we estimated a value of 13.3. We note that this value is highly consistent with experimentally derived ones [55].

In the case of white blood cells, estimated turnover rates vary widely, as pointed out by Tomasetti and Vogelstein [1]. Hematopoietic stem cells have been reported to divide every 15 days [56], but also every 57 days [57]. Moreover, hematopoietic progenitors are known to divide much more frequently: for instance, inactivated neutrophils, the major component of white blood cells, have an average lifespan of only 4 days [58] in line with other estimates [53]. Thus, overall, we assigned a turnover rate for blood tissue of 10 days, leading to an *IR* estimate for blood of 365/10 = 36.5.

### HypoClock solo-WCGW analysis

In analogy to epiTOC/epiTOC2, we decided to construct a mitotic-like clock based on the 6214 solo-WCGWs with representation on Illumina 450k/EPIC beadarrays, as derived by Berman and colleagues [17]. However, because a large fraction of the 6214 solo-WCGWs were not uniformly methylated across fetal tissue types (using Illumina 450k DNAm data from the SCM2 [45]), in order to avoid confounding by cell type heterogeneity as much as possible, we also restricted to a subset of 678 solo-WCGWs which did exhibit uniformly high methylation across 10 fetal tissue types (SCM2 data) [45] and cord blood [46]. Using the same underlying mathematical model of DNAm transmission between cell generations, one can derive an approximate anti-correlative measure for the total number of stem cell divisions per stem cell in a tissue, denoted by *HypoScore*, by taking the average DNAm over these 678 CpGs. We note that this is directly analogous to our previous epiTOC model, where we took an average over 385 PRC2-marked CpGs [15]. By analogy with epiTOC, the *HypoScore* in sample *s* of tissue type *x* must satisfy the following relation:

$$ 1- HypoScore\left(s,x\right)\sim A(s)R\left(s,x\right) $$

Observe that since the *HypoScore* is defined in terms of the average DNAm over solo-WCGWs, a decrease in the score must reflect increased deviations with age, which is why in the above equation we subtract the score from 1. It follows that the age-adjusted *HypoScore*, *HypoScore [AgeAdj]*, obtained by first dividing by the chronological age of the individual, and then taking the median estimator, should yield an anti-correlative measure of the intrinsic rate of stem cell division in the tissue, i.e.,:

$$ 1- HypoScore\left[ AgeAdj\right](x)={\left\langle \frac{1- HypoScore\left(s,x\right)}{Age(s)}\right\rangle}_s\sim IR(x) $$

where <*>_*s*_ denotes the median over all samples. As with epiTOC2, in datasets where chronological age was not available, we used Horvath’s epigenetic clock to obtain DNAm-based surrogates [59].

Of note, because the age-adjusted *HypoScore* is derived from taking the average DNAm level over the 678 solo-WCGWs and then dividing by age, it does not yield a direct estimate of the stem cell division rate, but only an anti-correlative estimate. It is therefore justified to rescale the age-adjusted *HypoScore* by a common scale factor, in order to preserve the original dynamic range of the *HypoScore* values. Assuming the vector of *HypoScore* values is denoted by *hypoSC*.*v* and the corresponding vector of age-adjusted *HypoScore* values is denoted by *hypoSCaa*.*v*, then the overall transformation used is

$$ 1- hypoSC.v\to \max \left(1- hypoSC.v\right)-\gamma \max (hypoSCaa.v)+\gamma\ hypoSCaa.v $$

where *γ =* (*max*(*hypoSC*.*v*)*-min*(*hypoSC*.*v*))*/*(*max*(*hypoSCaa*.*v*)*-min*(*hypoSCaa*.*v*)). Importantly, this same linear transformation must be applied to all samples over which the resulting values are being compared. For instance, in the cross-tissue analyses, the transformation is performed over all tissues together, to allow meaningful cross-tissue comparison.

### Normal adjacent and cancer Illumina 450k datasets from TCGA

We downloaded and processed level 3 Illumina 450k and RNA-SeqV2 data from the TCGA [49], as described by us previously [60]. In total, we considered 16 cancer types for which corresponding *IR* estimates in normal tissue could be found: BLCA (bladder urothelial carcinoma, nN = 19, nC = 204), BRCA (breast invasive carcinoma, nN = 81, nC = 652), COAD (colon adenocarcinoma, nN = 38, nC = 272), ESCA (esophageal carcinoma, nN = 15, nC = 126), HNSC (head and neck squamous cell carcinoma, nN = 45, nC = 402), KIRC (kidney renal cell carcinoma, nN = 160, nC = 299), KIRP (kidney renal papillary carcinoma, nN = 45, nC = 196), LIHC (liver hepatocellular carcinoma, nN = 47, nC = 176), LSCC (lung squamous cell carcinoma, nN = 41, nC = 275), LUAD (lung adenocarcinoma, nN = 32, nC = 399), PAAD (pancreatic adenocarcinoma, nN = 10, nC = 146), PRAD (prostate adenocarcinoma, nN = 48, nC = 278), READ (rectal adenocarcinoma, nN = 7, nC = 95), THCA (thyroid carcinoma, nN = 53,nC = 489), and STAD (stomach adenocarcinoma, nN = 2, nC = 393).

### Normal (non-adjacent) tissue DNAm datasets

We obtained Illumina 450k DNAm data from studies profiling normal tissue, not adjacent to cancer. Briefly, we obtained normal samples from the lung (*n* = 21) [36], breast (*n* = 171: 50 derived from [37] and 121 from [61]), oral/buccal (*n* = 790) [36], liver (*n* = 26) [62], stomach (*n* = 61) [24], colon (*n* = 8) [63], skin (*n* = 19) [64], esophagus (*n* = 52) [28], and whole blood (*n* = 487: 335 from Liu et al. [65] and 152 from Teschendorff et al. [36]). In all cases, we used the normalized data as described in our previous publications, or were normalized de novo using the same procedure as in these previous studies, i.e., using minfi [66] and BMIQ [67]. Further details can be found in the “Normalization of Illumina 450k/EPIC data” section.

### WGBS data from purified blood cell subtypes

We obtained normalized whole-genome bisulfite sequencing (WGBS) data representing 6 purified blood cell subtypes (neutrophils, monocytes, natural killer cells, CD4+ and CD8+ T cells, and B cells) from each of 3 donors, from Farlik et al. [68] (GEO: GSE87196). Because read-depth and coverage was low, in order to be able to call differential methylated cytosines between blood cell subtypes over a reasonable number of sites, we pooled data from the 3 donors together. Specifically, we only retained CpGs with at least 5 mapped reads in at least 1 donor. For these CpGs, we pooled total and methylated reads from all 3 donors, to define a DNAm beta value. For each pairwise comparison of blood cell subtypes (a total of 15 comparisons), we then defined DMCs as those with an absolute DNAm difference of 0.2 or greater. The total number of CpGs for which differential methylation analysis was performed were 408,011 (B vs. CD4T), 452,798 (B vs. CD8T), 640,384 (B vs. Monoc), 182,472 (B vs. NK), 560,658 (B vs. Neu), 502,878 (CD4T vs. CD8T), 716,763 (CD4T vs. Monoc), 195,686 (CD4T vs. NK), 624,790 (CD4T vs. Neu), 800,905 (CD8T vs. Monoc), 215,183 (CD8T vs. NK), 695,393 (CD8T vs. Neu), 289,639 (Monoc vs. NK), 1,004,611 (Monoc vs. Neu), and 262,921 (NK vs. Neu).

### Fetal tissue DNAm sets

We obtained and normalized Illumina 450k data from the Stem-Cell Matrix Compendium-2 (SCM2) [45], as described by us previously [15]. There were a total of 37 fetal tissue samples encompassing 10 tissue types (stomach, heart, tongue, kidney, liver, brain, thymus, spleen, lung, adrenal gland). We also obtained and normalized Illumina 450k data from 15 cord blood samples [46]. In all cases, data was normalized as described for the other studies.

### Purified blood cell subtype Illumina 450k DNAm sets

We used Illumina 450k data of purified CD4+ T cell (*n* = 214) and monocyte samples (*n* = 1202), as generated by the MESA study [69], and of CD8+ T cells (*n* = 98) [70]. These datasets were downloaded from GEO (GSE56046 and GSE56581), and intra-array normalized with BMIQ, as described by us previously [71]. We also analyzed Illumina 450k DNAm data of matched neutrophils, monocytes, and T cells from 139 individuals, generated as part of BLUEPRINT [72], previously normalized by us in [73].

### Precancerous Illumina 450k DNAm datasets

We analyzed two Illumina 450k DNAm datasets, one profiling normal gastric mucosa (*n* = 61), mild intestinal metaplasia (*n* = 22), and intestinal metaplasia (*n* = 108) [24], and another profiling normal colon (*n* = 8) and colorectal adenomas (*n* = 39) [63]. For the gastric dataset, idat files were downloaded from GEO (GSE103186) and processed with *minfi*. Probes with over 99% coverage were kept and missing values imputed using *impute* R-package [74]. Subsequently, data was intra-array normalized with BMIQ [67], resulting in a final normalized data matrix over 482,975 CpGs and 191 samples. For the colon dataset, idat files were downloaded from ArrayExpress (E-MTAB-6450) and processed with *minfi*. Only probes with 100% coverage were kept. Subsequent data was intra-array normalized with BMIQ, resulting in a normalized data matrix over 485,512 CpGs and 47 samples.

### Analysis of cell type heterogeneity

Using WGBS data from purified blood cell subtypes, we called DMCs between each pair of blood cell subtypes, as described in earlier section. We then computed the fraction of solo-WCGWs, PMD solo-WCGWs that were DMCs, and compared this fraction to that obtained using all CpGs. We used a one-tailed Fisher test to assess statistical significance that the fraction for solo-WCGWs is higher than for the full set.

In the case of SCM2 data, we performed hierarchical clustering of the 37 fetal samples over the corresponding solo-WCGW or epiTOC2/epiTOC sites. In the case of the large purified blood cell subtype datasets, we performed PCA over the corresponding sites and correlated each of the significant components of variation with chronological age. The number of significant components was estimated using random matrix theory [75].

### Normalization of Illumina 450k/EPIC data

All Illumina 450k/EPIC datasets analyzed in this study have been normalized using a common procedure, as described by us previously in the respective publications. Very briefly, idat files were processed using *minfi* Bioconductor package [66], without background correction and using Illumina’s definition of beta value. Background correction increases technical variance despite removing bias in the U and M channels and is therefore a procedure which is only advisable if U and M values are used directly for inference (e.g., as in CNV estimation [76]). Probes with detection *P* values above 0.05 were flagged, and for studies with less than 25 samples, only probes with no missing values were retained for further analysis. For larger-scale studies, where imputation with *k*-nearest neighbors (*k* = 5) [74] is meaningful, we allowed probes to contain at most 1% missing values. To correct for type 2 probe bias, we used our BMIQ normalization procedure [67]. Inter-sample variation was assessed using a SVD, where we correlated inferred components of variation to technical factors, including beadchip, plate, and position. Batch effects, if present, were removed with COMBAT [77].

### Code availability

An R-script, *epiTOC2*.*R*, implementing epiTOC2, and the list of 163 epiTOC2 CpGs and 678 constitutively methylated solo-WCGWs are available as an .Rd object, *dataETOC2*.*Rd*, all freely available from 10.5281/zenodo.2632938

### Data availability

The main Illumina DNA methylation datasets used here are freely available from public repositories, including GEO (www.ncbi.nlm.nih.gov/geo), ArrayExpress (www.ebi.ac.uk/arrayexpress), and EGA (www.ebi.ac.uk/ega/home) (see Online Methods for relevant references). Details: Hannum (656 whole blood, GEO: GSE40279); MESA (214 purified CD4+ T cells and 1202 Monocyte samples, GEO: GSE56046 and GSE56581); Tserel (98 CD8+ T cells, GEO: GSE59065); BLUEPRINT (139 matched CD4+ T cells, Monocytes and Neutrophils, EGA: EGAS00001001456); Liu (335 whole blood, GEO: GSE42861); Gastric tissue (191 normal and metaplasia, GEO: GSE103186); Colon tissue (47 normal and adenoma, ArrayExpress: E-MTAB-6450); Breast Erlangen (50 normal, GEO: GSE69914); Breast2 (121 normal, GEO: GSE101961); Liver (26 normal, GEO: GSE61258); Skin (19 epidermal non-sun exposed, GEO: GSE51954); Esophagus (52 normal, GEO: GSE104707); SCM2 (37 fetal tissue samples, GEO: GSE31848); Cord-Blood (15 samples, GEO: GSE72867). Blueprint-WGBS (18 samples, GEO: GSE87196). TCGA data was downloaded from https://gdc.cancer.gov. The DNAm dataset in buccal cells as well as 152 whole blood samples from the NSHD is available by submitting data requests to mrclha.swiftinfo@ucl.ac.uk; see full policy at http://www.nshd.mrc.ac.uk/data.aspx. Managed access is in place for this 69-year-old study to ensure that use of the data are within the bounds of consent given previously by participants and to safeguard any potential threat to anonymity since the participants are all born in the same week.

## Results

### Construction of epiTOC2

**Fig. 1:**
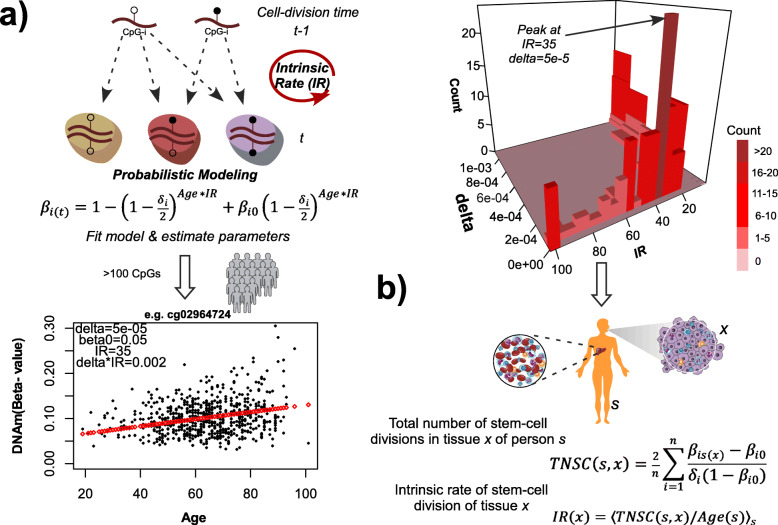
The epiTOC2 model. **a** epiTOC2 is based on a DNAm transmission model between cell-generations, from which a mathematical expression can be derived that relates the measured DNAm fraction at a given CpG site *i* at cell division time *t*, *β*_*i*(*t*)_, to the site’s de novo methylation probability *δ*_*i*_ and ground-state (i.e., fetal stage) methylation *β*_*i0*_, as well as the chronological age of the individual and the intrinsic annual rate of stem cell division per stem cell (*IR*), which is CpG independent. These parameters can be estimated using a large cohort of healthy individuals for which DNAm at specific CpG sites (the epiTOC2-CpGs) has been measured in a common tissue (we do this for blood). An example of an epiTOC2 CpG demonstrating a reasonably good fit to the model is shown together with the estimated parameters. The 3-dimensional bar chart displays the number of epiTOC2-CpGs from a total of 163 (*z*-axis, count) with particular estimated de novo methylation probabilities (*x*-axis, delta *δ*) and intrinsic stem cell division rate estimates (*y*-axis, *IR*), as derived by fitting the epiTOC2 model to 656 whole blood samples. The bar chart reveals a clear mode at *IR = 35*, which we take as an estimate of the intrinsic rate of stem cell division of blood. **b** With the *δ*_*i*_ and *β*_*i0*_ parameters estimated in **a**, which are assumed to be approximately tissue independent, one can estimate the total number of stem cell divisions in any given tissue *x* of an individual *s*, *TNSC*(*s*,*x*), from the measured DNAm fraction over the 163 epiTOC2 CpGs. If the age of the person is known, one can estimate the average lifetime rate of stem cell division per stem cell of the person, and for large cohorts of healthy individuals, the intrinsic rate of tissue type *x* can be estimated by taking the average or median over all samples *s*

Here, we build and improve on previous studies [15, 16, 43] to construct a novel mitotic model called epiTOC2 that can directly estimate the cumulative number of stem cell divisions per stem cell in a tissue. epiTOC2 uses a formal mathematical model for DNA methylation transmission between cell generations [43] at specific CpGs that are unmethylated across many different fetal tissue types, thus allowing definition of a proper cell type independent “ground-state” (Methods). From this model, we first derived a mathematical expression relating the fraction of cells methylated at one of these CpG sites *i* in a given sample, to the total number of stem cell divisions (*TNSC*) at cell division time “*t*”, and to parameters reflecting the site-specific probability of de novo methylation *δ*_*i*_ and ground-state methylation (i.e., at fetal stage) *β*_*i0*_ (Methods, Fig. 1a). We note that *TNSC* factorizes as the product of the chronological age of the sample and an average lifetime intrinsic rate (*IR*) of stem cell division, i.e., TNSC = Age*IR (Fig. 1a, Methods). By applying non-linear least squares to DNAm data from a large cohort of healthy individuals of known ages in a common tissue type *x*, we can reliably estimate the above parameters for hundreds of CpGs that satisfy our selection criteria, as well as estimating the intrinsic stem cell division rate *IR*(*x*) of the tissue (Methods, Fig. 1a). Using whole blood DNAm profiles for over 656 healthy individuals [47], we estimated the *δ*_*i*_ and *β*_*i0*_ parameters for 163 eligible CpG loci (Methods, Additional file 1: Tables S1-S2, Fig. S1), as well as estimating the *IR* of stem cell division in blood to be approximately 35 divisions per year (Fig. 1a, Methods), i.e., cell renewal approximately every 10 days, consistent with literature-based estimates [1]. To demonstrate that the *IR* estimate is not an artifact, we applied the same fitting procedure to data generated via a simulation model, where parameters including *IR* are known in advance, and which incorporates a realistic age-dependent heteroscedastic noise term (Methods). Resulting parameter estimates correlated well with the true values, thus validating our procedure and confirming the identifiability of the model parameters, including *IR* (Additional file 1: Fig. S2). We also obtained similar *IR* and *δ*_*i*_ estimates when applying the same procedure to 335 whole blood samples from an independent healthy cohort [65], validating the robustness and stability of the estimation (Additional file 1: Fig. S3a-b). *δ*_*i*_ parameter estimates across loci were also correlated between different blood cell subtypes (Additional file 1: Fig. S3c), as estimated by applying our fitting procedure to DNAm profiles of purified monocytes and CD4+ T cells [69]. Thus, assuming the parameters *δ*_*i*_ and *β*_*i0*_ are to a large extent tissue- and cell-type independent, by measuring DNA methylation across the same 163 loci in an independent sample *s* from an arbitrary tissue type *x*, one can estimate the total number of stem cell divisions *TNSC*(*s*,*x*) (Methods, Fig. 1b) as

$$ \mathrm{TNSC}\left(s,x\right)=\frac{2}{n}\sum \limits_{i=1}^n\frac{\beta_{is(x)}-{\beta}_{i0}}{\delta_i\left(1-{\beta}_{i0}\right)} $$

where *n =* 163 and *β*_*is*(*x*)_ is the methylation beta value at CpG *i* in sample *s* of tissue type *x*. We verified that the 163 epiTOC2 CpGs exhibit highly stable and uniform unmethylation across different fetal tissue types (Additional file 1: Fig. S4a) and that clustering of these fetal samples over the sites did not segregate samples according to tissue type (Additional file 1: Fig. S5a), confirming that *β*_*i*0_ is largely independent of tissue type [15, 30]. Of note, the above formula means that epiTOC2 subsumes our previous epiTOC model [15] (Methods).

Importantly, if the chronological age of the individual *s* is known, the above formula allows estimation of the average lifetime rate of stem cell division in tissue type *x* of individual *s*, denoted by *R*(*s*,*x*) as *R*(*s*,*x*) *= TNSC*(*s*,*x*)*/A*(*s*). For sufficiently large cohorts of healthy individuals for which DNAm has been profiled in the same tissue type *x*, we can estimate the intrinsic rate of stem cell division in that tissue type, *IR*(*x*), by taking the median or average of *R*(*s*,*x*) over all samples *s* (Methods, Fig. 1b).

### Validation of epiTOC2 in normal tissue

**Fig. 2:**
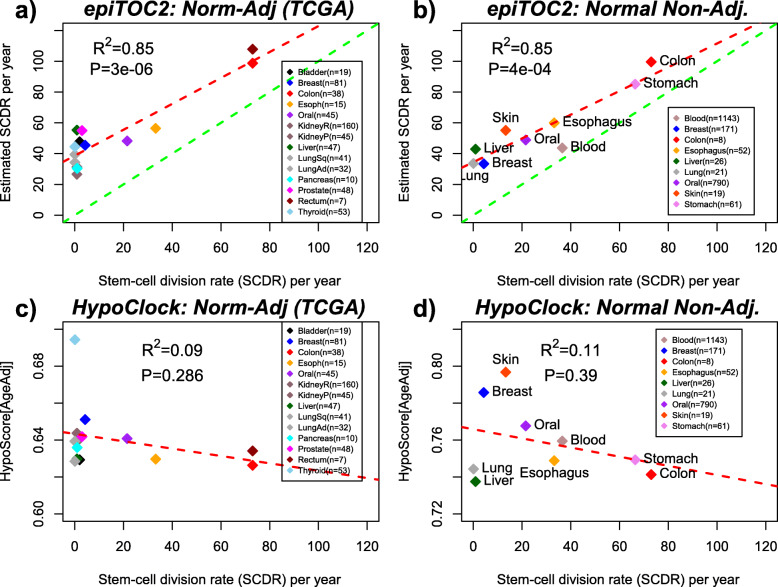
Cross-tissue comparison of mitotic clock rate estimates with experiment-derived stem cell division rates. **a** Scatterplot of the estimated epiTOC2 stem cell division rate per stem cell per year (*y*-axis, automatically age-adjusted) versus the corresponding literature based estimate for normal tissues (*x*-axis) profiled as part of the TCGA, as indicated. *R*^2^ and *P* value from a linear regression are given. For each normal tissue, we provide the number of independent samples, and each datapoint represents the median value over these samples. **b** As **a**, but now for epiTOC2 stem cell division rate estimates obtained in normal tissue samples not adjacent to cancer. **c**, **d** As **a**, **b** but now for the HypoClock and the age-adjusted HypoScore derived from it

Using epiTOC2, we obtained *IR* estimates in the normal adjacent samples from The Cancer Genome Atlas (TCGA) [78] (Methods) and compared these to independent stem cell division rate estimates for bladder, breast, colon, esophagus, oral, kidney, liver, lung, pancreas, prostate, rectum, and thyroid tissue, as obtained from the literature (Methods) [1, 50]. This revealed a remarkably good correlation between epiTOC2 and literature-based estimates (*R*^2^ = 0.85, PCC = 0.92, *P* = 3e−6, Fig. 2a). Because tissues exhibit a wide range of *IR* values, we also assessed the correlation in a log basis, which effectively assigns more weight in the regression to tissues with lower turnover rates: although the *R*^2^ value dropped, we still observed a statistically significant correlation (*R*^2^ = 0.51, PCC = 0.71, *P* = 0.004, Additional file 1: Fig. S6a). Interestingly, epiTOC2 estimates were always larger than the ones derived from the literature (Fig. 2a). A plausible explanation for this could be that the normal tissue found adjacent to cancer is already characterized by significant DNAm alterations and a marginally increased mitotic-rate, as suggested by previous studies [15, 37].

To investigate this further, we collected independent DNAm profiles from normal tissue specimens that were not adjacent to cancer and from individuals that were deemed healthy, whenever this was possible (Methods, Additional file 1: Table S3). Importantly, we included more tissue types with high or intermediate cell turnover rates, including stomach, skin, and blood. In this second analysis, we also observed a strong correlation between epiTOC2 and literature-based *IR* estimates (*R*^2^ = 0.85, PCC = 0.92, *P* = 4e−4, Fig. 2b), which remained significant using a log basis (*R*^2^ = 0.55, PCC = 0.73, *P* = 0.023, Additional File 1: Fig. S6b). In this second analysis, epiTOC2 estimates were also consistently larger than the literature-based ones (Fig. 2b), suggesting that the higher estimates from epiTOC2 in the normal adjacent tissues from TCGA is not due to their proximity to cancer cells.

### Validation of epiTOC2 in inflammatory and precancerous conditions

**Fig. 3:**
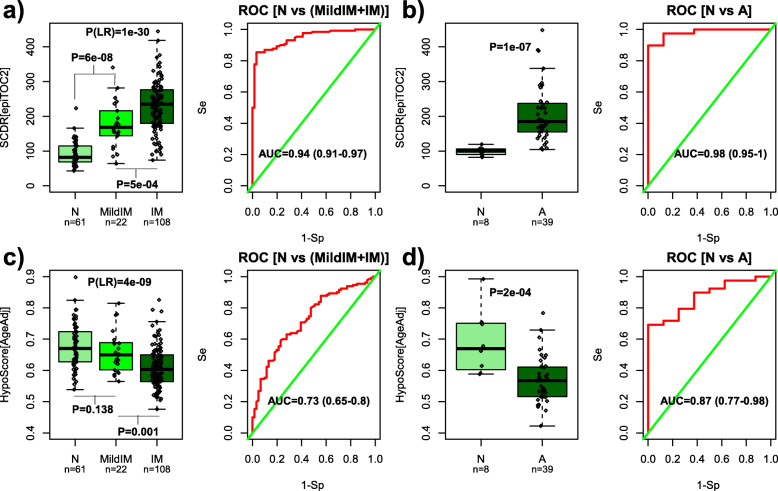
Epigenetic mitotic clock rate estimates in precancerous inflammatory conditions. **a** Left panel: Estimated stem cell division rate (SCDR) from epiTOC2 (*y*-axis) against the progression stage in gastric metaplasia (*x*-axis, N = normal gastric mucosa, MildIM = mild intestinal metaplasia, IM = advanced intestinal metaplasia). The number of samples in each group is indicated. *P* value at top, denoted by P(LR), is derived from a linear regression of SCDR against progression stage. Other *P* values derive from one-tailed Wilcoxon-rank sum tests comparing neighboring progression stages. Right panel: ROC curve, AUC value, and 95% confidence interval for SCDR discriminating normal from metaplasia (MildIM+IM). **b** As **a**, but now for a study profiling DNAm in normal colon (N) and colorectal adenoma (A). ROC and AUC show ability of estimated SCDR to discriminate N from A. **c**, **d** As **a**, **b** but for the HypoClock model

To further test the validity of epiTOC2, we applied it to a condition characterized by chronic inflammation, since this is known to increase the turnover rate of a tissue [5, 79]. We investigated this in the context of gastrointestinal metaplasia, where inflammation (driven by factors such as *H*. *pylori* infection) is known to drive metaplasia and the risk of gastric cancer [6, 24]. Using epiTOC2, we estimated the rate of stem cell division in 61 normal gastric specimens, in 22 exhibiting a mild form of intestinal metaplasia (“MildIM”), and in 108 with high-risk intestinal metaplasia (“IM”), all for which Illumina DNAm 450k profiles had been generated [24] (Methods). Confirming the validity of epiTOC2, the estimated stem cell division rate was markedly increased in the MildIM and IM samples (linear regression *P* = 1e−30, Fig. 3a), allowing almost perfect discrimination of normal and metaplasia (MildIM+IM) samples (AUC = 0.94 (95% CI 0.91–0.97) (Fig. 3a).

We also investigated whether epiTOC2 would predict an increased mitotic rate in colorectal adenoma, a well-known precursor lesion of colorectal cancer [63]. Although less frequently associated with inflammatory conditions such as ulcerative colitis and Crohn’s disease [80], its incidence increases with age [81], suggesting that it may also be driven by increased cellular turnover. The stem cell division rate (SCDR) estimated by epiTOC2 was significantly higher in the adenomas compared to normal colon (Wilcox rank-sum test *P* = 1e−7, Fig. 3b) and highly accurate in discriminating normal from adenoma tissue (AUC = 0.98 (95% CI 0.95–1), Fig. 3b). We point out that all these associations are already automatically adjusted for age, since the estimated cell division rates were obtained by dividing the estimated total number of stem cell divisions by chronological age or, if not available, by DNAm-based chronological age surrogates obtained from Horvath’s epigenetic clock [59], which we point out is not a mitotic clock [15, 59]. We further note that while similar findings were obtained using our previous epiTOC model (Additional file 1: Fig. S7), only epiTOC2 yields direct mitotic count estimates. For instance, using epiTOC2, we have estimated that in mild intestinal metaplasia each adult stem cell undergoes 168 divisions per year, in comparison to the 82 average for the normal gastric samples, a near 2-fold increase. In colorectal adenoma, the median SCDR was 185, compared to 99.6 for normal colon, a ratio also close to 2. Of note, this ratio is smaller than the drift ratio of 3 to 4 observed when comparing colon carcinoma to normal colon tissue [27], consistent with colon carcinoma being more proliferative than adenoma.

### A considerable fraction of solo-WCGW sites are cell type-specific markers

**Fig. 4:**
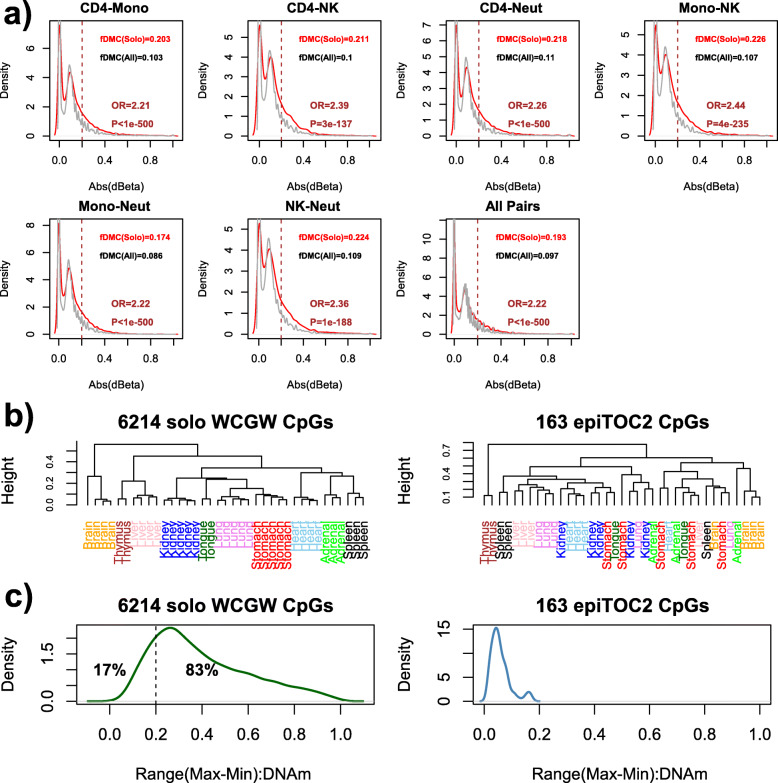
Solo-WCGWs are confounded by cell type heterogeneity. **a** Using WGBS data encompassing 6 blood cell subtypes from each of 3 donors, we display the density distributions of absolute differences in DNAm between corresponding pairs of blood cell-subtypes (first 7 panels), as indicated. In gray, we depict the distribution for all CpGs with at least 5 reads mapped in at least one of the donors, whereas in red, we depict the corresponding distribution for the subset of solo-WCGWs. In each panel, we give the fraction of CpGs with absolute DNAm difference larger than 0.2 (vertical dashed line), the threshold used to declare a differentially methylated cytosine (DMC). We also provide the corresponding odds ratio (OR) and associated one-tailed *P* value from a Fisher test. The last panel summarizes the result for all 15 pairs combined. **b** Unsupervised clustering of 37 fetal tissue samples representing 10 tissue types, over the 6214 HM450k PMD solo-WCGW CpGs (left panel) and over the 163 epiTOC2 CpGs (right panel). **c** Distribution of the range in DNAm exhibited by the 6214 HM450k PMD solo-WCGWs (left panel) and by the 163 epiTOC2 CpGs, as assessed over the 37 fetal tissue samples from 10 different tissue types

In order to benchmark epiTOC2, we compared it to a recently proposed mitotic model based on taking the average methylation over a large number of solo WCGW CpGs [17]. However, as with the epiTOC2 sites, we first decided the check the implicit and unproven assumption that solo-WCGWs are not confounded by cell type heterogeneity [41, 42]. Using whole-genome bisulfite-sequencing (WGBS) data from 6 purified blood cell subtypes from each of 3 different donors (thus matched for age and genotype) [68], we observed that solo-WCGWs were approximately twice more likely to be differentially methylated between blood cell subtypes compared to a randomly selected set of CpGs (Fig. 4a, Additional file 1: Fig. S8, Methods), a result which was robust to the choice of thresholds for calling significant differential DNAm (Additional file 1: Fig. S9). Of note, this bias of solo-WCGWs towards being cell type-specific DNAm markers became stronger when restricting to solo-WCGWs that mapped to PMDs (Additional file 1: Fig. S10). Overall, we estimated that at least 20 to 30% of solo-WCGWs may be prone to confounding by cell type heterogeneity, in contrast to only 10% when considering all CpGs in the genome (Fig. 4a, Additional file 1: Figs. S8–10).

We confirmed the cell type specificity of solo-WCGWs by an independent analysis, whereby we clustered the same set of 37 fetal samples from 10 different tissue types considered earlier [45], but now, over the 6214 solo-WCGWs with representation on Illumina HM450k beadarrays. This resulted in perfect segregation of samples according to tissue type, in stark contrast to the substantial inter-mixing of tissue types when clustering over epiTOC2 CpGs (Fig. 4b). To quantify this further, we observed that the DNAm values of the 6214 solo-WCGWs across the 37 fetal samples were not stably methylated (Additional file 1: Fig. S4b) and that 83% of the solo-WCGWs exhibited DNAm changes across fetal tissue types that were greater than 20% (Fig. 4c). Approximately 28% of the solo-WCGWs exhibited DNAm differences greater than 50%. We note that such large changes in DNAm between fetal tissue types would be hard to attribute to differences in mitotic age, and are much more likely to reflect cell type-specific DNAm, as indeed cell type composition is generally the main driver of DNAm variation across normal samples [41]. Remarkably, even when restricting to a subset of 678 solo-WCGWs which exhibited uniformly high methylation across all fetal samples, these samples still segregated according to tissue type (Additional file 1: Fig. S5b). Thus, solo-WCGWs appear to be highly cell type specific.

### Hypomethylation at solo-WCGWs does not correlate with mitotic age

We next compared epiTOC2 to a mitotic clock based on taking the average methylation over the 678 solo-WCGWs that are least confounded by cell type heterogeneity (termed “HypoClock,” Methods), which is thus mathematically analogous to our previous epiTOC model based on taking the average methylation at specific PRC2-marked CpGs [15]. The mitotic age score from the HypoClock (termed “HypoScore”) did not exhibit an association with stem cell division rate estimates across normal tissues, even after adjustment for chronological age (Fig. 2c, d, Additional file 1: Fig. S11). To explore this further, we reasoned that top principal components (PCs) derived from a PCA over solo-WCGWs and over a large collection of healthy individuals representing different age groups, all profiled in the same tissue type, should correlate with chronological age, since in such cohorts mitotic age should be proportional to chronological age. In order to reduce the confounding effect of cell type heterogeneity, we once again restricted analysis to the subset of 678 solo-WCGWs and focused on genome-wide DNAm studies profiling purified cell types. Using DNAm data from Blueprint, encompassing Illumina 450k data for monocytes, neutrophils, and T cells from 139 individuals [72], we confirmed that top PCs over the solo-WCGWs did not correlate with age, in stark contrast to those obtained from epiTOC2 CpGs (Additional file 1: Fig. S12a). We analyzed an additional 3 large DNAm datasets profiling purified monocytes (*n* = 1202), CD4+ (*n* = 214), and CD8+ T cells (*n* = 98) [69, 70] with near identical results (Additional file 1: Fig. S12b-c). Similar results were obtained when correlating the average DNAm over the respective epiTOC2 or solo-WCGW sites to chronological age (Additional file 1: Fig. S13). Moreover, average DNAm levels over solo-WCGWs, as assessed in 8 different normal adjacent tissue cohorts from TCGA, did not anti-correlate with chronologic age, with the exception of colon (Additional file 1: Fig. S14). In contrast, the cumulative total number of stem cell divisions estimated with epiTOC2 did correlate with age for 5 different tissue types (Additional file 1: Fig. S15). Of note, the only 3 tissues for which there was no association of epiTOC2 with chronological age were breast, prostate, and thyroid, i.e., hormone-responsive tissues (Additional file 1: Fig. S16), suggesting that for these tissues external hormonal factors may play a more important role than chronological age in dictating their mitotic age. In summary, all these data suggest that solo-WCGWs, unlike epiTOC2 CpGs, do not track mitotic age in a normal physiological context.

### epiTOC2 identifies precancerous lesions better than HypoClock

Next, we assessed the ability of HypoClock to discriminate precancerous lesions from normal tissue, using the previous DNAm data from gastrointestinal metaplasias and colorectal adenomas. Although the HypoClock-score was able to discriminate metaplasias from normal gastric mucosa (Fig. 3c) and colorectal adenomas from normal colon (Fig. 3d), discriminatory power was much reduced compared to epiTOC2 (Fig. 3a, b). We observed that these results held true in other tissue types (Additional file 1: Figs. S16–17). For instance, epiTOC2 could discriminate normal breast tissue adjacent to breast cancer from normal breast from healthy women with an AUC = 0.65 (Wilcox test *P* = 0.007), whereas HypoClock could not (AUC = 0.48, Wilcox *P* = 0.38) (Additional File 1: Fig. S16). In lung tissue, epiTOC2 could discriminate normal lung tissue from lung carcinoma in-situ (LCIS) with an AUC = 0.76 (Wilcox test *P* = 0.005), whereas with HypoClock the association was only marginal (AUC = 0.66, Wilcox *P* = 0.07) (Additional file 1: Fig. S17). Interestingly, using DNAm data from the TCGA to compare normal and cancer tissue revealed a similar pattern, with both clocks being predictive, but with epiTOC2 a much stronger and consistent discriminator than HypoClock (Additional file 1: Figs. S18-S19).

## Discussion

The results presented here provide strong evidence that dynamic DNAm changes can be used to approximate the mitotic age of a tissue. The formulation of a concrete mathematical model for how DNAm is transmitted through cell generations allowed us to derive a novel formula relating measured DNAm over specific CpGs to the underlying cumulative number of stem cell divisions. The resulting epiTOC2 model yielded stem cell division rate estimates in normal tissues that correlated remarkably well (Pearson correlation ~ 0.92) with their expected turnover rate, as determined by independent experimental methods. While this strong correlation was observed within each of two separate collections of normal tissues, epiTOC2 estimates were also consistently higher than experimentally derived ones. This offset however was relatively small, and the epiTOC2 estimates were of the same order of magnitude than the experimental ones, which attests to the overall validity of the epiTOC2 model. Furthermore, a relatively small offset is inevitable and could easily arise due to a number of factors. For instance, tissues like lung or colon contain a substantial amount of stromal cells, notably immune cells (ICs) [82]. Thus, measuring an average DNAm over all the cell types in a tissue would correspond to taking an average over the division rates of each major cell type in the tissue. While this may explain why *IR* estimates were higher for low-turnover tissues like lung or bladder, it is unclear why for a very high turnover tissue like colon, the estimated *IR* value was not lower. Other factors that make a direct comparison of absolute division rates difficult include (i) selection bias in sample collection, since for most epithelial tissues, normal specimens are more likely to be collected from high-risk individuals; (ii) the assumption that the stem cell division rate is constant throughout life, when for most tissues the rate is significantly higher during development and childhood; and (iii) the relative simplicity of the epiTOC2 model, as the model ignored stem cell pool size and the hierarchical organization of tissues which include various progenitor cell populations. Nevertheless, epiTOC2 was further validated in the context of chronic inflammation within colon and gastric tissue, as well as in high-risk breast and lung tissue samples, thus confirming its potential use for cancer risk prediction applications.

Importantly, our results have also revealed a profound difference depending on the specific CpGs used to construct the mitotic clock model. epiTOC2 is based on hypermethylation of PRC2-marked sites that are unmethylated across a wide range of different fetal tissue types, whereas the HypoClock model is based on solo-WCGWs, which are normally methylated at a fetal stage and which would gradually lose methylation due to incomplete maintenance in late-replicating regions. While HypoClock is supported by an attractive mechanistic model, the data presented in this work suggests that this mode of methylation loss may only operate or is only significant in highly proliferative cells, but not in stem cells undergoing normal tissue turnover. Indeed, unlike epiTOC2, HypoClock estimates did not correlate with known stem cell division rates of normal tissues and did not correlate with chronological age in large cohorts representing healthy individuals. In addition, HypoClock performed consistently worse than epiTOC2 at identifying precancerous lesions and cancer itself. These results indicate that solo-WCGWs do not faithfully track cumulative cell division numbers in normal adult tissue.

While these findings appear to contradict those of Berman and colleagues [17], we offer a number of plausible explanations as to why solo-WCGWs are not suitable for measuring mitotic age in normal adult tissues. First, using WGBS data we have seen that approximately 20–30% of the 3.7 million solo-WCGWs considered by Berman and colleagues [17] are subject to confounding by cell type heterogeneity. Thus, comparing average DNAm over solo-WCGWs must be interpreted with caution, as DNAm differences could arise because of variations in cell type proportions and not because of differences in mitotic age. Indeed, variations in the cellular milieu between fetal samples and those from later developmental stages could easily drive the observed gradual decrease in average DNAm levels over solo-WCGWs in early development [17]. Although Berman and colleagues did not address the confounding posed by cell type heterogeneity, we did address it here by focusing on a subset of solo-WCGWs which were least affected by cell type heterogeneity. However, even for this subset, estimated mitotic rates did not correlate with known estimates across normal tissues. In this regard, we emphasize that a key advantage of the epiTOC2 model is that it is based on CpGs which, at the fetal stage, are not cell type specific, thus avoiding the confounding effects of cell type heterogeneity.

A second reason why in normal (adult) tissue HypoClock performs substantially worse than epiTOC2 could be the selection procedure of solo-WCGWs: these sites were identified by comparing normal to cancer tissue across many different cancer-types [17] and therefore may not be suitable for tracking cell division during normal tissue turnover. Indeed, cancer cells are under high replicative stress, which may explain the observed enrichment of solo-WCGWs in late-replicating regions [17]. Assuming cell type heterogeneity is not a confounder, this would also explain why solo-WCGWs hypomethylation is observed in early development when cells are also under high replicative stress. Thus, HypoClock is not a good mitotic model for normal adult tissues, but it can correctly predict an increased mitotic count in high-replicative stress conditions such as cancer and early development.

A potential caveat to our interpretation is that most of our analyses were restricted to a reduced subset of solo-WCGWs with representation on methylation beadarrays. However, all the key analyses by Berman and colleagues [17] that were performed in normal adult cells were also restricted to the same HM450k beadarrays, with the authors not concluding that this restriction was a limitation. All HypoClock results presented here were also independent of whether we focused on the 6214 HM450k solo-WCGWs, or the subset of 678 solo-WCGWs that were least confounded by cell type heterogeneity. While we cannot exclude the existence of a small subset of solo-WCGWs which may track mitotic age independently of cell type composition and other confounders, the most likely explanation for the discrepant results obtained in adult normal tissue is that specific results presented in Zhou et al. [17] are indeed confounded by cell type heterogeneity or batch effects [83]. Based on our data, we would argue that averaging DNAm over 3.7 million solo- WCGWs (or 1.8 million PMD solo-WCGWs) to approximate mitotic age is unjustified, not only because of cell type heterogeneity and the potentially biased selection method, but also because averages taking over such large numbers of CpGs could be easily driven by different subsets in different conditions. Such an assay would also appear infeasible in practice. Instead, cumulative hypermethylation at a relatively small number of 163 epiTOC2 CpGs appears to be a much better and robust mitotic age clock for adult tissues, and so we propose that measurement of DNAm at these sites in adult pre-neoplastic lesions, potentially in serum cell-free DNA [19], could provide the basis for building feasible pre-diagnostic or cancer risk assays.

## Conclusions

In summary, we have presented a novel epigenetic mitotic clock epiTOC2 that can yield approximate estimates of the cumulative number of stem cell divisions in normal adult tissue. epiTOC2 outperforms epigenetic mitotic clocks based on hypomethylation of solo-WCGWs within PMDs and late-replicating regions.

## Supplementary information

**Additional file 1: Figure S1.** Estimation of epiTOC2 parameters. **Figure S2.** Identifiability of parameter estimation procedure. **Figure S3.** Stability of parameter estimation procedure. **Figure S4.** Density distribution of DNA methylation beta-values for mitotic clock CpG sites across fetal tissue samples. **Figure S5.** Unsupervised clustering of fetal tissue samples over mitotic clock CpG sites. **Figure S6.** Correlation between epiTOC2 and literature-based stem-cell division rates in a logged basis. **Figure S7.** epiTOC in inflammatory and precancerous conditions. **Figure S8.** Solo-WCGWs are twice more likely to be cell-type specific markers compared to randomly selected CpGs. **Figure S9.** Solo-WCGWs are more likely to be cell-type specific markers compared to randomly selected CpGs: robustness to choice of significance threshold. **Figure S10.** Solo-WCGWs mapping to PMDs are almost three times more likely to be cell- type specific markers compared to randomly selected CpGs. **Figure S11.** No association between HypoClock score and literature-based stem-cell division rates in a logged basis. **Figure S12.** Associations of epiTOC2-CpGs and solo-WCGWs with chronological age in blood cell subtypes. **Figure S13.** Associations of average DNAm over epiTOC2 and PMD solo-WCGWs with age in purified cell-types. **Figure S14.** No consistent anti-correlation between HypoClock score and chronological age in normal-adjacent tissue from TCGA. **Figure S15.** Correlation of epiTOC2 scores with chronological age in normal-adjacent tissue from TCGA. **Figure S16.** Comparison between epiTOC2 and HypoClock in breast tissue. **Figure S17.** Comparison between epiTOC2 and HypoClock in lung tissue. **Figure S18.** epiTOC2 predicts increased mitotic rate in cancer. **Figure S19.** Associations of HypoClock-score with normal/cancer status in samples from TCGA. **Table S1:** Estimated epiTOC2 parameters. **Table S2.** Final epiTOC2 parameters. **Table S3.** Summary of normal-tissue (non-TCGA) collection.

## Data Availability

The main Illumina DNA methylation 450k datasets used here are freely available from public repositories, including GEO (www.ncbi.nlm.nih.gov/geo), ArrayExpress (www.ebi.ac.uk/arrayexpress), and EGA (www.ebi.ac.uk/ega/home) (see Methods for relevant references). Details: Hannum (656 whole blood, GEO: GSE40279) [47]; MESA (214 purified CD4+ T cells and 1202 Monocyte samples, GEO: GSE56046 and GSE56581) [69]; Tserel (98 CD8+ T cells, GEO: GSE59065) [70]; BLUEPRINT (139 matched CD4+ T cells, Monocytes and Neutrophils, EGA: EGAS00001001456) [72]; Liu (335 whole blood, GEO: GSE42861) [65]; Gastric tissue (191 normal and metaplasia, GEO: GSE103186) [24]; Colon tissue (47 normal and adenoma, ArrayExpress: E-MTAB-6450) [63]; Breast Erlangen (50 normal, GEO: GSE69914) [37]; Breast2 (121 normal, GEO: GSE101961) [61]; Liver (26 normal, GEO: GSE61258) [62]; Skin (19 epidermal non-sun exposed, GEO: GSE51954) [64]; Esophagus (52 normal, GEO: GSE104707) [28]; SCM2 (37 fetal tissue samples, GEO: GSE31848) [45]; Cord blood (15 samples, GEO: GSE72867) [46]. TCGA data was downloaded from https://gdc.cancer.gov. The DNAm dataset of buccal cells, as well as 152 whole blood samples from the NSHD was published previously [36] and is available by submitting data requests to mrclha.swiftinfo@ucl.ac.uk; see full policy at http://www.nshd.mrc.ac.uk/data.aspx. Managed access is in place for this 69-year-old study to ensure that use of the data are within the bounds of consent given previously by participants and to safeguard any potential threat to anonymity since the participants are all born in the same week. An R-script, *epiTOC2*.*R*, implementing epiTOC2, and the list of 163 epiTOC2 CpGs and 678 constitutively methylated solo-WCGWs are available as an .Rd object, *dataETOC2*.*Rd*, all freely available from 10.5281/zenodo.2632938
