# Supplementary material for: A comparison of epigenetic mitotic-like clocks for cancer risk prediction
Source: Genome Med. 2020 Jun 24;12:56. doi: 10.1186/s13073-020-00752-3 (PMC7315560; doi:10.1186/s13073-020-00752-3)
Supplement: Supplementary file 1 — Additional file 1: Figure S1. Estimation of epiTOC2 parameters. Figure S2. Identifiability of parameter estimation procedure. Figure S3. Stability of parameter estimation procedure. Figure S4. Density distribution of DNA methylation beta-values for mitotic clock CpG sites across fetal tissue samples. Figure S5. Unsupervised clustering of fetal tissue samples over mitotic clock CpG sites. Figure S6. Correlation between epiTOC2 and literature-based stem-cell division rates in a logged basis. Figure S7. epiTOC in inflammatory and precancerous conditions. Figure S8. Solo-WCGWs are twice more likely to be cell-type specific markers compared to randomly selected CpGs. Figure S9. Solo-WCGWs are more likely to be cell-type specific markers compared to randomly selected CpGs: robustness to choice of significance threshold. Figure S10. Solo-WCGWs mapping to PMDs are almost three times more likely to be cell- type specific markers compared to randomly selected CpGs. Figure S11. No association between HypoClock score and literature-based stem-cell division rates in a logged basis. Figure S12. Associations of epiTOC2-CpGs and solo-WCGWs with chronological age in blood cell subtypes. Figure S13. Associations of average DNAm over epiTOC2 and PMD solo-WCGWs with age in purified cell-types. Figure S14. No consistent anti-correlation between HypoClock score and chronological age in normal-adjacent tissue from TCGA. Figure S15. Correlation of epiTOC2 scores with chronological age in normal-adjacent tissue from TCGA. Figure S16. Comparison between epiTOC2 and HypoClock in breast tissue. Figure S17. Comparison between epiTOC2 and HypoClock in lung tissue. Figure S18. epiTOC2 predicts increased mitotic rate in cancer. Figure S19. Associations of HypoClock-score with normal/cancer status in samples from TCGA. Table S1: Estimated epiTOC2 parameters. Table S2. Final epiTOC2 parameters. Table S3. Summary of normal-tissue (non-TCGA) collection. [file 13073_2020_752_MOESM1_ESM.pdf]

Supplementary Information for "A comparison of epigenetic mitotic-like clocks for cancer risk prediction"

Andrew E. Teschendorff [1,2,*]

1. CAS Key Laboratory of Computational Biology, CAS-MPG Partner Institute for Computational Biology, Shanghai Institute of Nutrition and Health, Shanghai Institutes for Biological Sciences, University of Chinese Academy of Sciences, Chinese Academy of Sciences, 320 Yue Yang Road, Shanghai 200031, China.

2. UCL Cancer Institute, Paul O'Gorman Building, University College London, 72 Huntley Street, London WC1E 6BT, United Kingdom.

*Corresponding author: Andrew E. Teschendorff- andrew@picb.ac.cn

SUPPLEMENTARY FIGURES

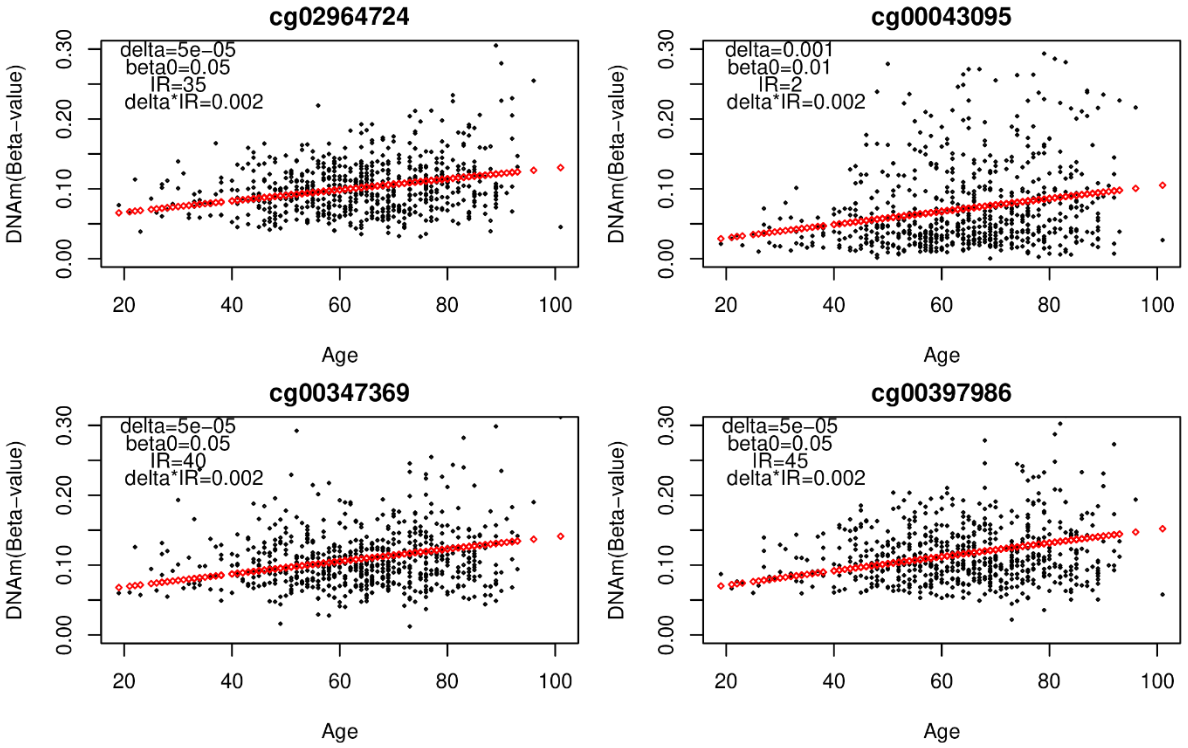

fig.S1: Estimation of epiTOC2 parameters. Example DNAm profiles for 4 epiTOC2 CpGs, as a function of chronological age in the Hannum et al 656 whole blood dataset [1]. All exhibit a significant correlation with age, and all satisfy the requirement that the product of de-novo methylation probability (*delta*) and the estimated intrinsic rate (*IR*) satisfies $delta*IR \geq 0.001$. Also shown are the estimated ground state DNAm values (*beta0*). We note that these profiles are for the initial fitting, and that subsequently all models are refitted with *IR=35,* since most of the 163 epiTOC2 CpGs yielded a common *IR* estimate of 35.

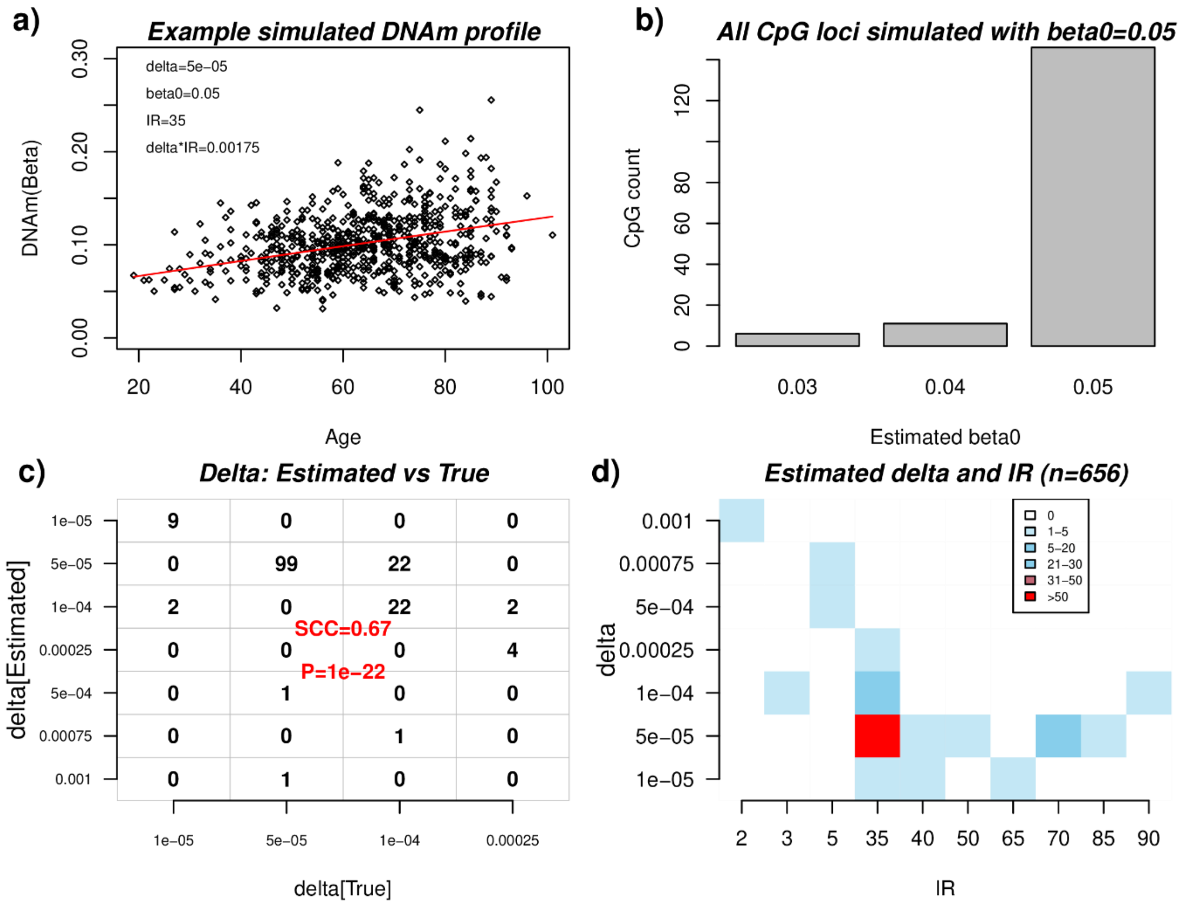

fig.S2: Identifiability of parameter estimation procedure. a) An example of a simulated DNAm profile for n=656 samples with an age-distribution as in Hannum et al, and with beta0=0.05, IR=35 and delta=5e-5. Red line shows best fit obtained via a non-linear least squares procedure. **b)** Estimated beta0 parameters for a simulation of 163 loci where all loci start out with beta0=0.05. **c)** Table count of estimated vs true delta parameters for the 163 loci. Statistical significance is estimated by computing a Spearman rank correlation coefficient (SCC) between the estimated and true delta parameters over the 163 loci. P-value of the Spearman test is shown. **d)** Heatmap displaying the counts of estimated delta-IR parameter combinations. Simulation was run with a true IR value of 35.

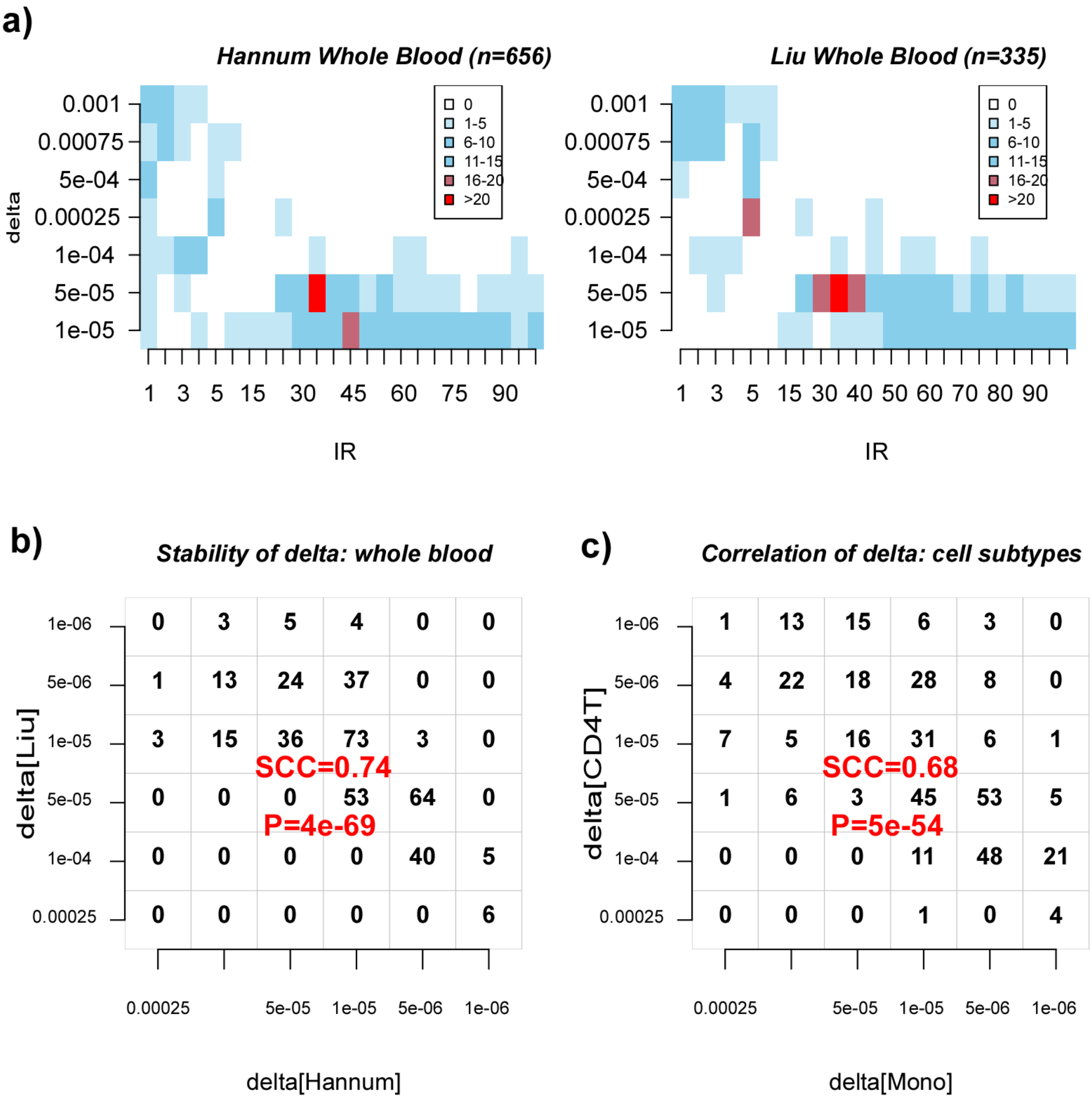

fig.S3: Stability of parameter estimation procedure

a) Count heatmap diagrams of the initial fitting of *delta* and *IR* in both Hannum et al and independently in the 335 whole blood control samples from Liu et al [2], in both cases revealing a maximum at *IR=35, delta=5e-5*. **b)** Table entries give the number of original PRC2-marked loci with particular *delta* parameter estimate combinations in Hannum and Liu whole blood cohorts. The *delta*-parameters were estimated using IR=35 in both cohorts, since this was the common mode in both cohort. SCC=Spearman rank Correlation Coefficient between the *delta* parameter estimates in the two cohorts, as evaluated over the loci, and P denotes the P-value derived from the Spearman rank correlation test. **c)** As b), but now comparing the *delta*-estimates derived from two purified blood cell subtype cohorts (Monocytes and CD4+ T-cells) from Reynolds et al [3]. These estimates were also obtained using IR=35, since once again this was the mode in both studies.

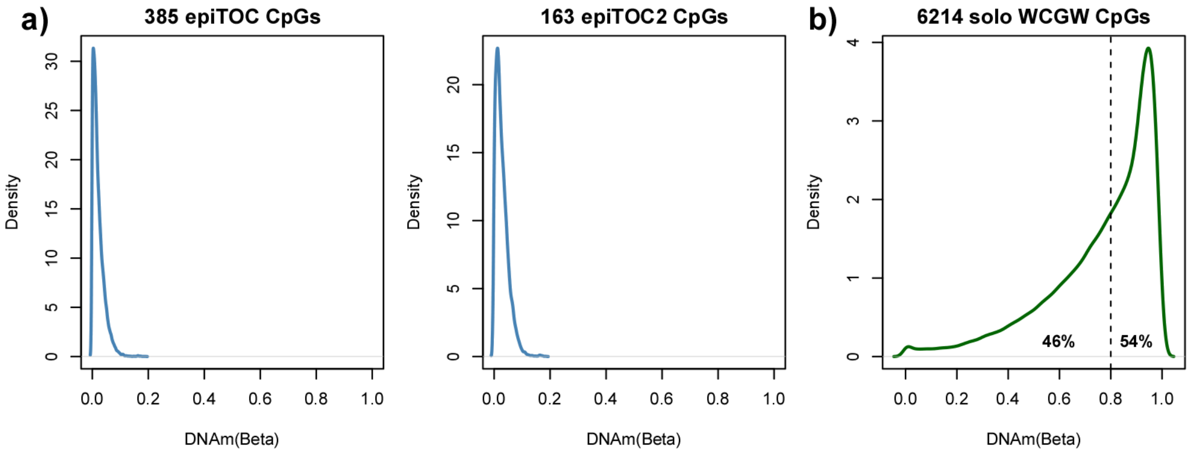

fig.S4: Density distribution of DNA methylation beta-values for mitotic clock CpG sites across fetal tissue samples. a) Density distribution of DNAm beta-values for the 385 epiTOC and 163 epiTOC2 Polycomb-Group Target promoter CpG sites across 37 fetal samples (encompassing 10 fetal tissue types) using the Infinium 450k data from the Stem-Cell Matrix Compendium-2 (SCM2) [4]. **b)** As a), but for the 6214 solo WCGW CpGs.

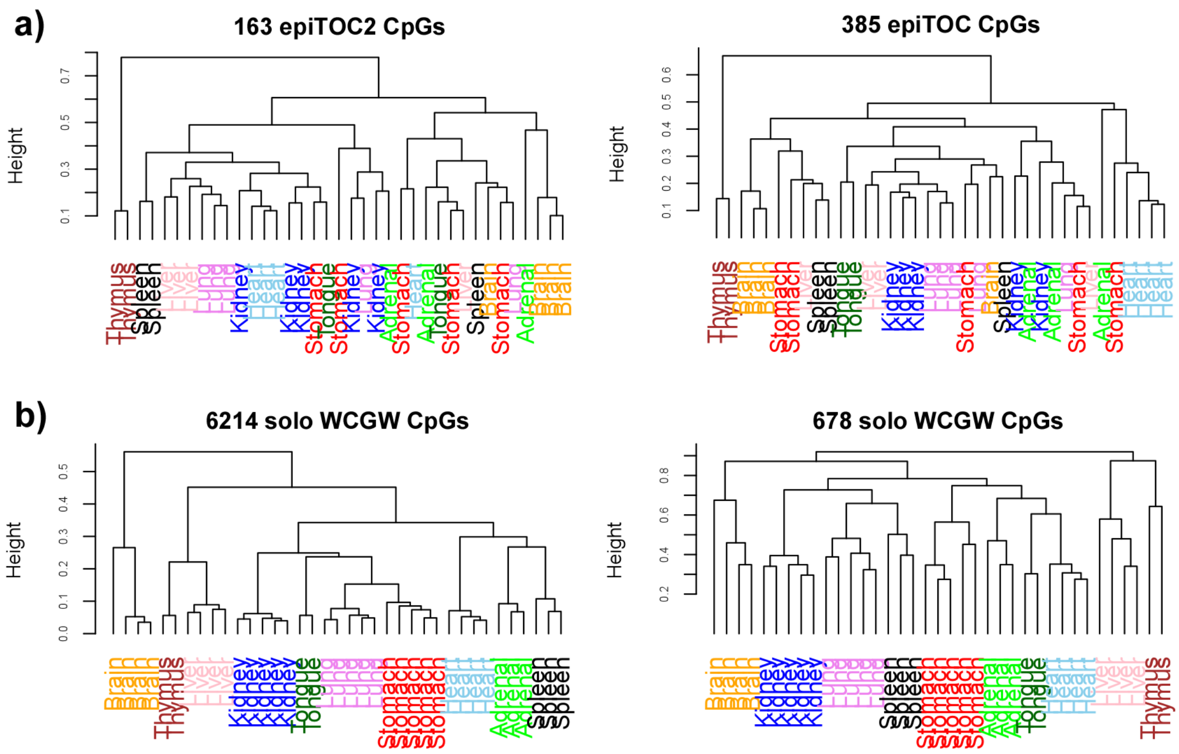

fig.S5: Unsupervised clustering of fetal tissue samples over mitotic clock CpG sites. Unsupervised hierarchical clustering of 37 fetal tissue samples from SCM2 [4] over 4 different subsets of mitotic clock CpGs: **a)** the 163 epiTOC2 CpGs and the 385 epiTOC CpGs [5]. **b)** the 6214 solo WCGW CpGs [6] and a subset of 678 solo WCGW CpGs that are fully methylated (beta > 0.8) across all 37 fetal samples.

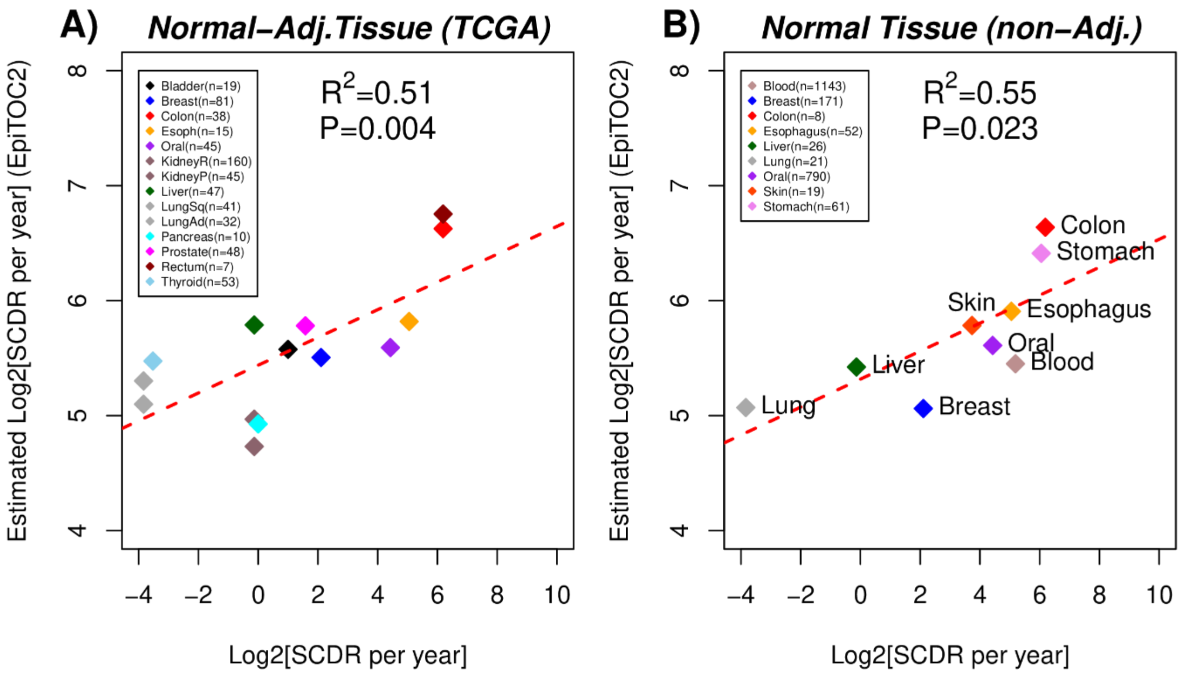

fig.S6: Correlation between epiTOC2 and literature-based stem-cell division rates in a logged basis. A) Scatterplot of the estimated epiTOC2 stem-cell division rate per stem-cell per year versus the corresponding literature based estimate for normal-tissues profiled as part of the TCGA, as indicated. The plot is shown on a log2-basis to highlight the differences between tissues with low to medium turnover. R^2 and P-value from a linear regression are given. For each normal tissue we provide the number of independent samples, and each datapoint represents the median value over these number of samples. **B)** As A), but now for epiTOC2 stem-cell division rate estimates obtained in normal tissue samples not adjacent to cancer.

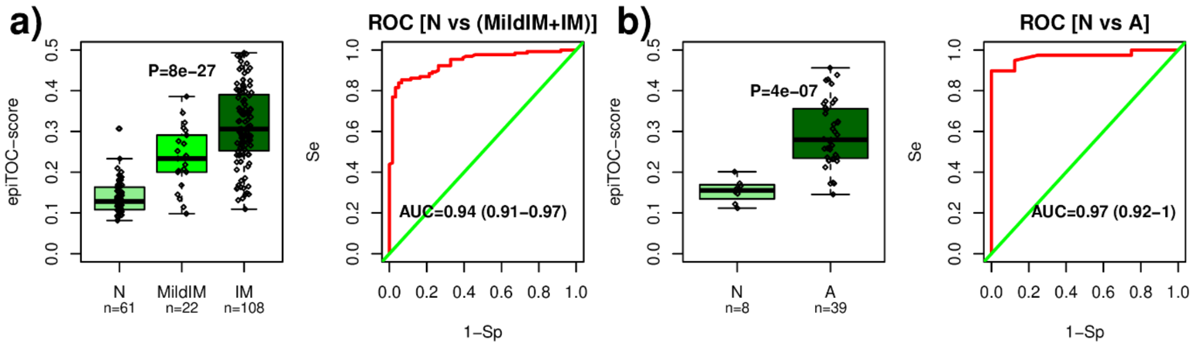

fig.S7: epiTOC in inflammatory and precancerous conditions. a) Left panel: The average DNAm of over epiTOC CpGs (epiTOC-score, y-axis) against the progression stage in gastric metaplasia (x-axis, N=normal gastric mucosa, MildIM=mild intestinal metaplasia, IM=advanced intestinal metaplasia). The number of samples in each group is indicated. P-value at top is derived from a linear regression of epiTOC-score against progression stage. **Right panel:** ROC curve, AUC value and 95% confidence interval for epiTOC-score discriminating normal from metaplasia (MildIM+IM). **b)** As a), but now for a study profiling DNAm in normal colon (N) and colorectal adenoma (A). ROC and AUC show ability of epiTOC-score to discriminate N from A.

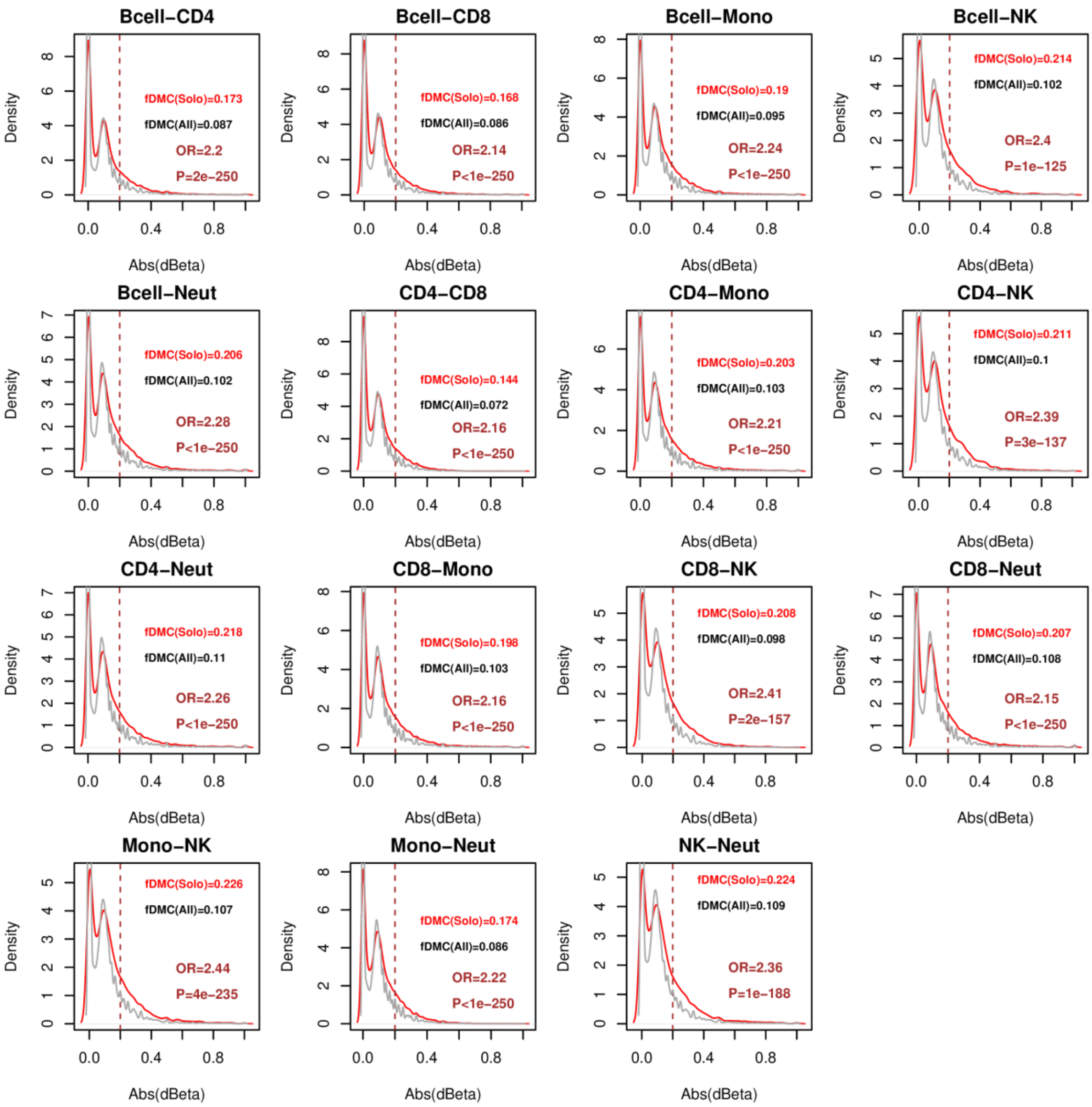

fig.S8: Solo-WCGWs are twice more likely to be cell-type specific markers compared to randomly selected CpGs. Using WGBS data encompassing 6 blood cell subtypes from each of 3 donors from Farlik et al [7], we display the density distributions of absolute differences in DNAm between corresponding pairs of blood cell-subtypes, as indicated. In grey we depict the distribution for all CpGs with at least 5 mappable reads in at least one of the donors, whereas in red we depict the corresponding distribution for the subset of solo-WCGWs. In each panel, we give the fraction of CpGs with absolute DNAm difference larger than 0.2 (vertical dashed line), the threshold used to declare a differentially methylated cytosine (DMC). We also provide the corresponding Odds Ratio (OR) and associated one-tailed P-value from a Fisher-test.

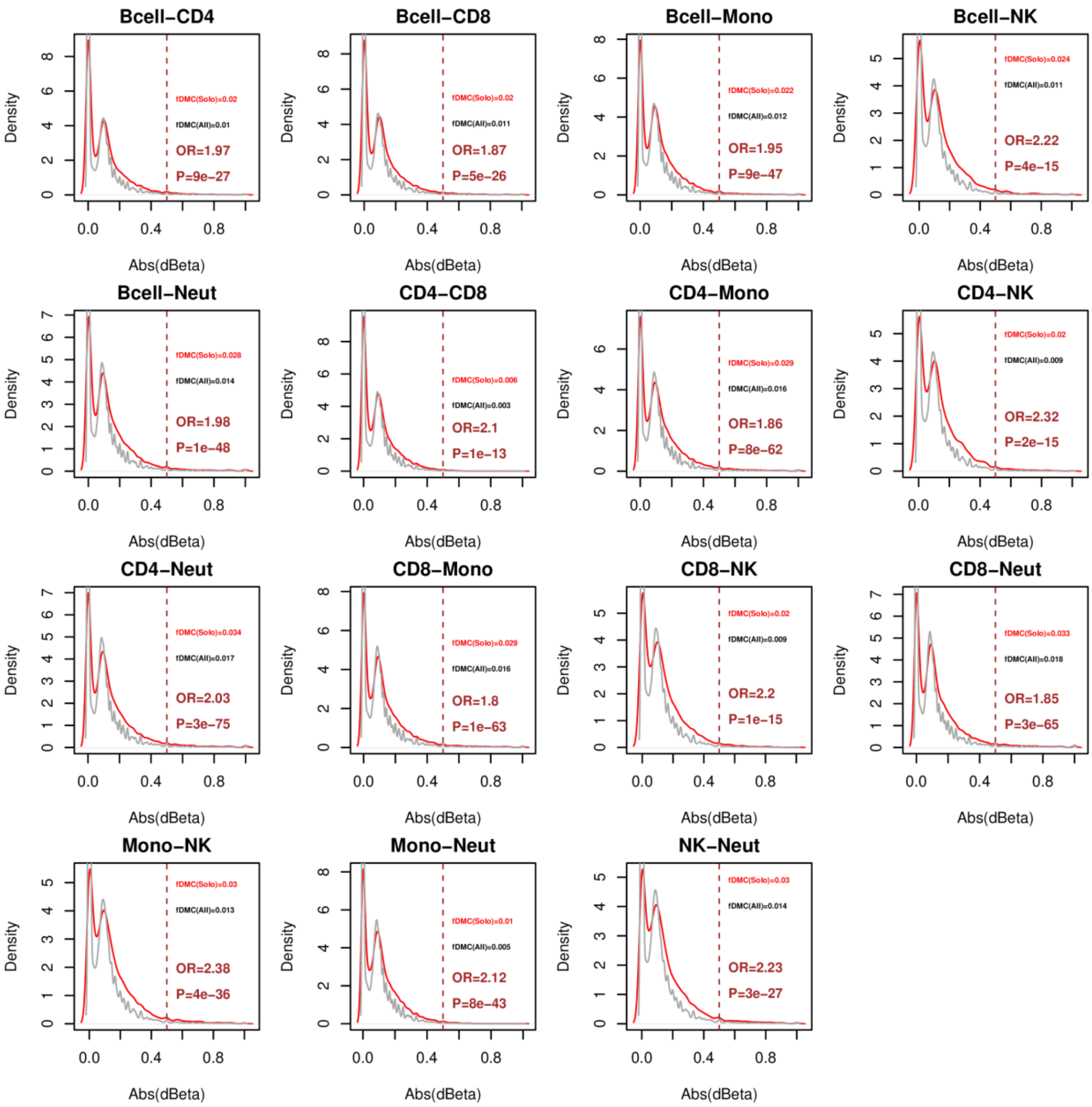

fig.S9: Solo-WCGWs are more likely to be cell-type specific markers compared to randomly selected CpGs: robustness to choice of significance threshold. Using WGBS data encompassing 6 blood cell subtypes from each of 3 donors from Farlik et al [7], we display the density distributions of absolute differences in DNAm between corresponding pairs of blood cell-subtypes, as indicated. In grey we depict the distribution for all CpGs with at least 5 mappable reads in at least one of the donors, whereas in red we depict the corresponding distribution for the subset of solo-WCGWs. In each panel, we give the fraction of CpGs with absolute DNAm difference larger than 0.5 (vertical dashed line), the threshold used to declare a differentially methylated cytosine (DMC). We also provide the corresponding Odds Ratio (OR) and associated one-tailed P-value from a Fisher-test.

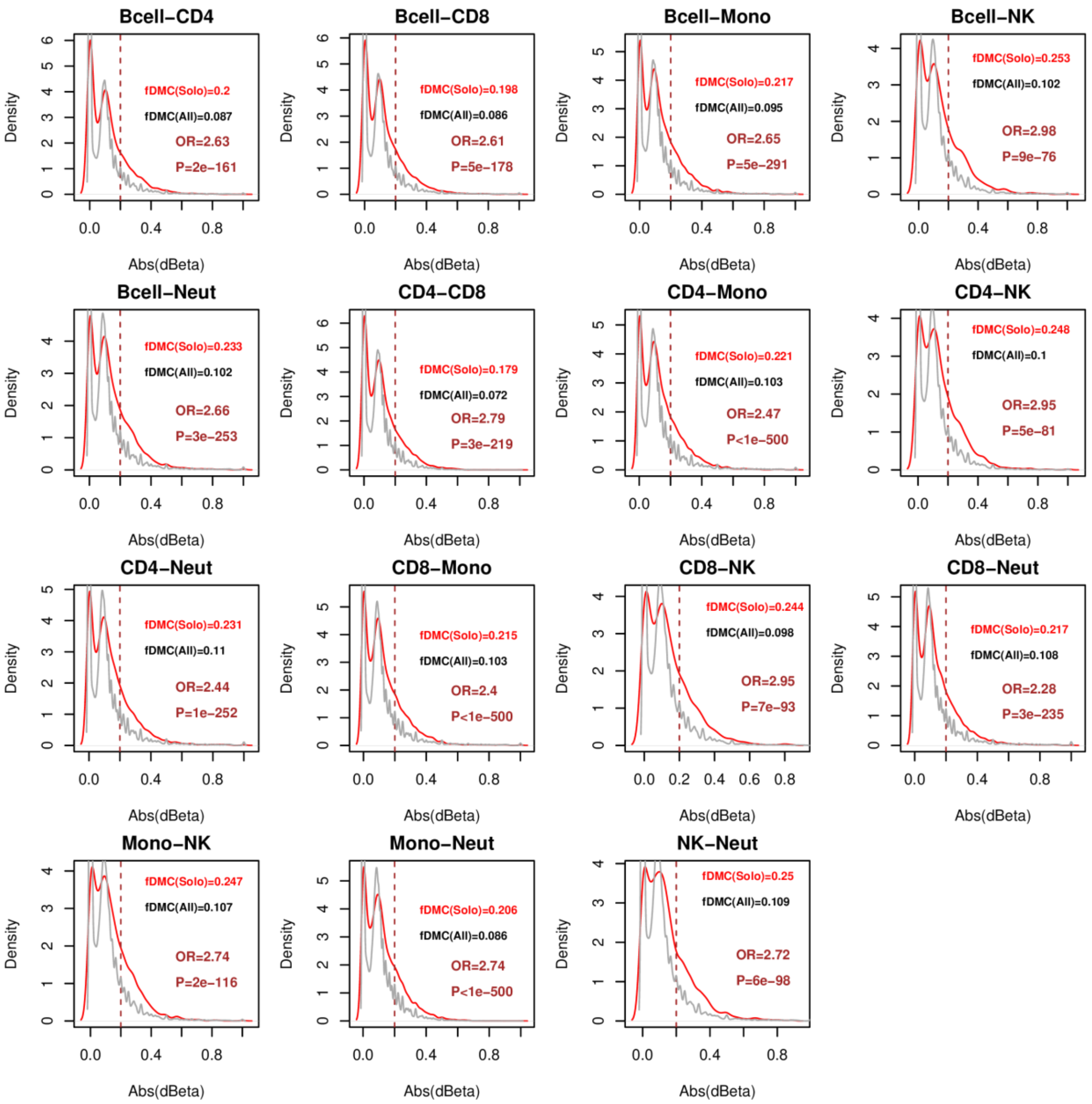

fig.S10: Solo-WCGWs mapping to PMDs are almost three times more likely to be cell-type specific markers compared to randomly selected CpGs. Using WGBS data encompassing 6 blood cell subtypes from each of 3 donors from Farlik et al [7], we display the density distributions of absolute differences in DNAm between corresponding pairs of blood cell-subtypes, as indicated. In grey we depict the distribution for all CpGs with at least 5 mappable reads in at least one of the donors, whereas in red we depict the corresponding distribution for the subset of PMD solo-WCGWs. In each panel, we give the fraction of CpGs with absolute DNAm difference larger than 0.2 (vertical dashed line), the threshold used to declare a differentially methylated cytosine (DMC). We also provide the corresponding Odds Ratio (OR) and associated one-tailed P-value from a Fisher-test.

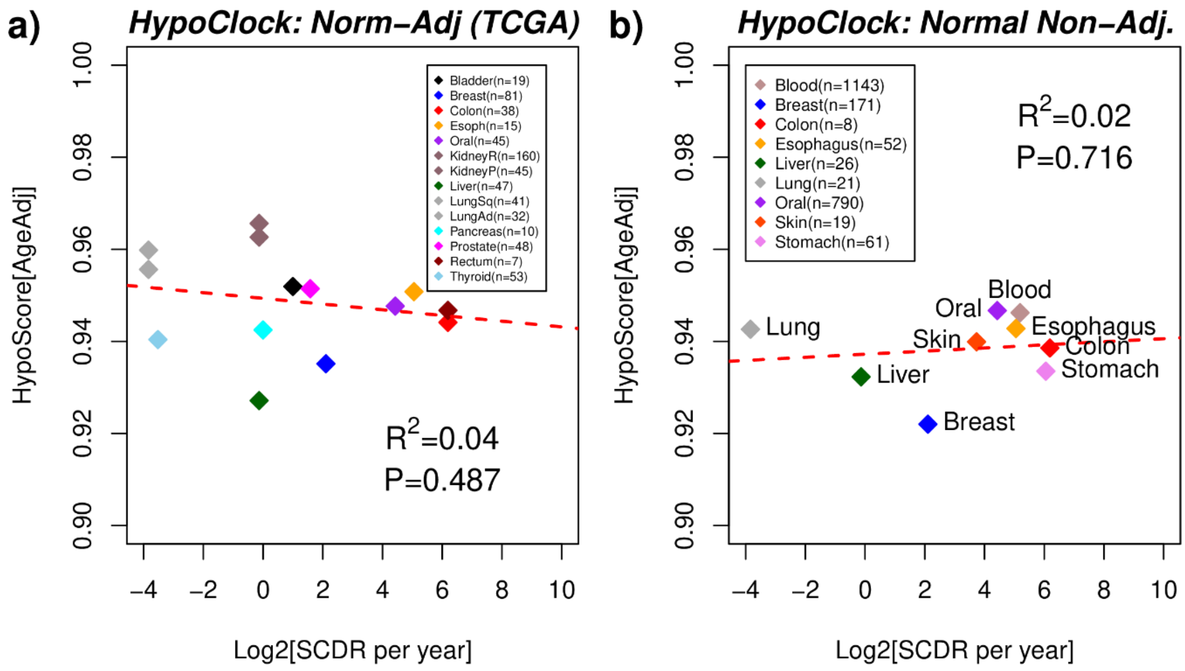

fig.S11: No association between HypoClock score and literature-based stem-cell division rates in a logged basis. a) Scatterplot of the HypoClock score adjusted for chronological age [y-axis] vs the independent stem-cell division rate per year in a logged basis [x-axis] for each normal-adjacent tissue of the TCGA. Values represent the median for each tissue, where the number of samples in each tissue is given in legend. R^2 value and P-value of a linear regression are given. **b)** As a), but now for all the normal tissue samples, not adjacent to cancer, as derived from independent DNAm studies.

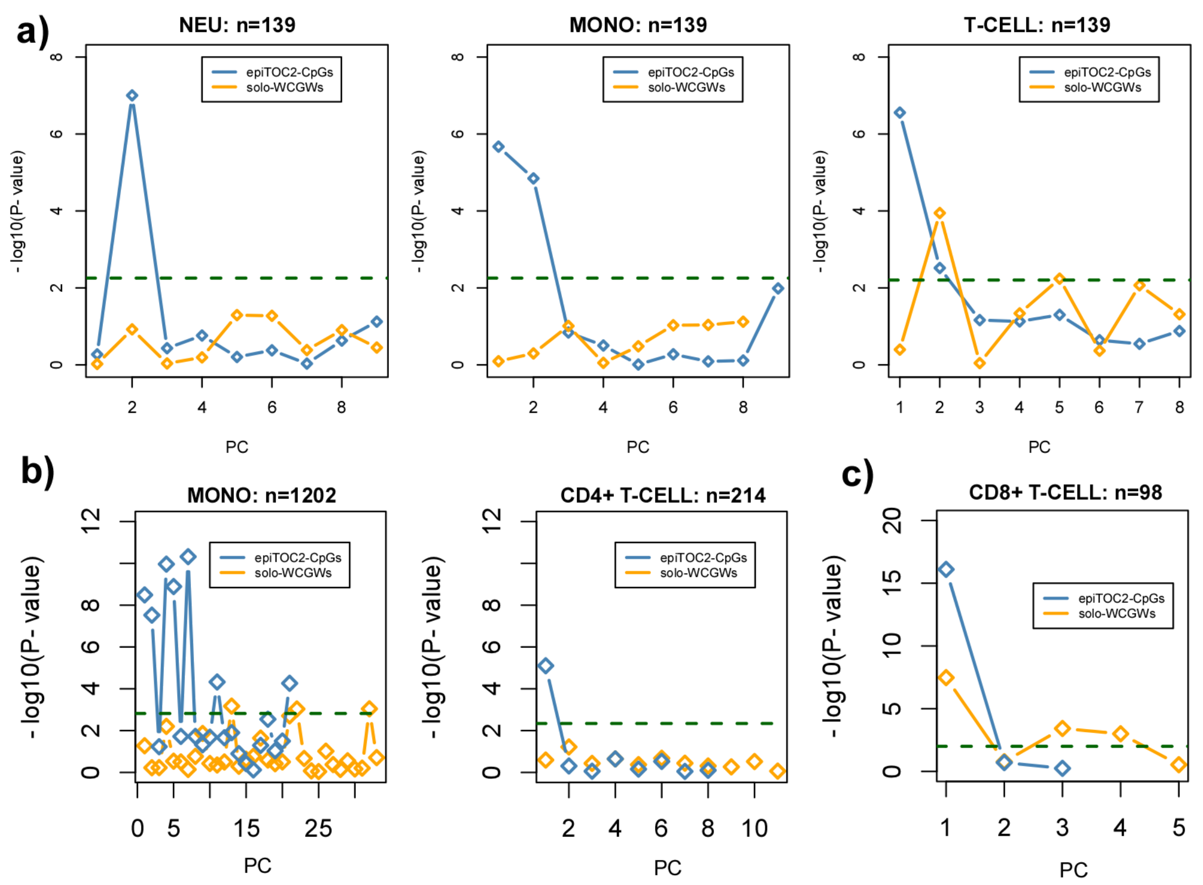

fig.S12: Associations of epiTOC2-CpGs and solo-WCGWs with chronological age in blood cell subtypes. a) Plots of significance ($-\log_{10}$[P-value], y-axis) of association of top-PCs (x-axis) with chronological age in 3 different purified blood cell subtype DNAm datasets (NEU=neutrophils, MONO=monocytes, T-cell=CD4+ T-cells) from the Blueprint consortium [8], with the number of samples indicated on top. PCs were derived from a PCA on the reduced DNAm data matrices obtained by restricting to either the 163 epiTOC2 CpGs, or the 678 solo-WCGWs, as indicated. **b-c)** As a), but now for the purified monocytes and CD4+ T-cells from Reynolds et al [3] and the CD8+ T-cells from Tserel et al [9].

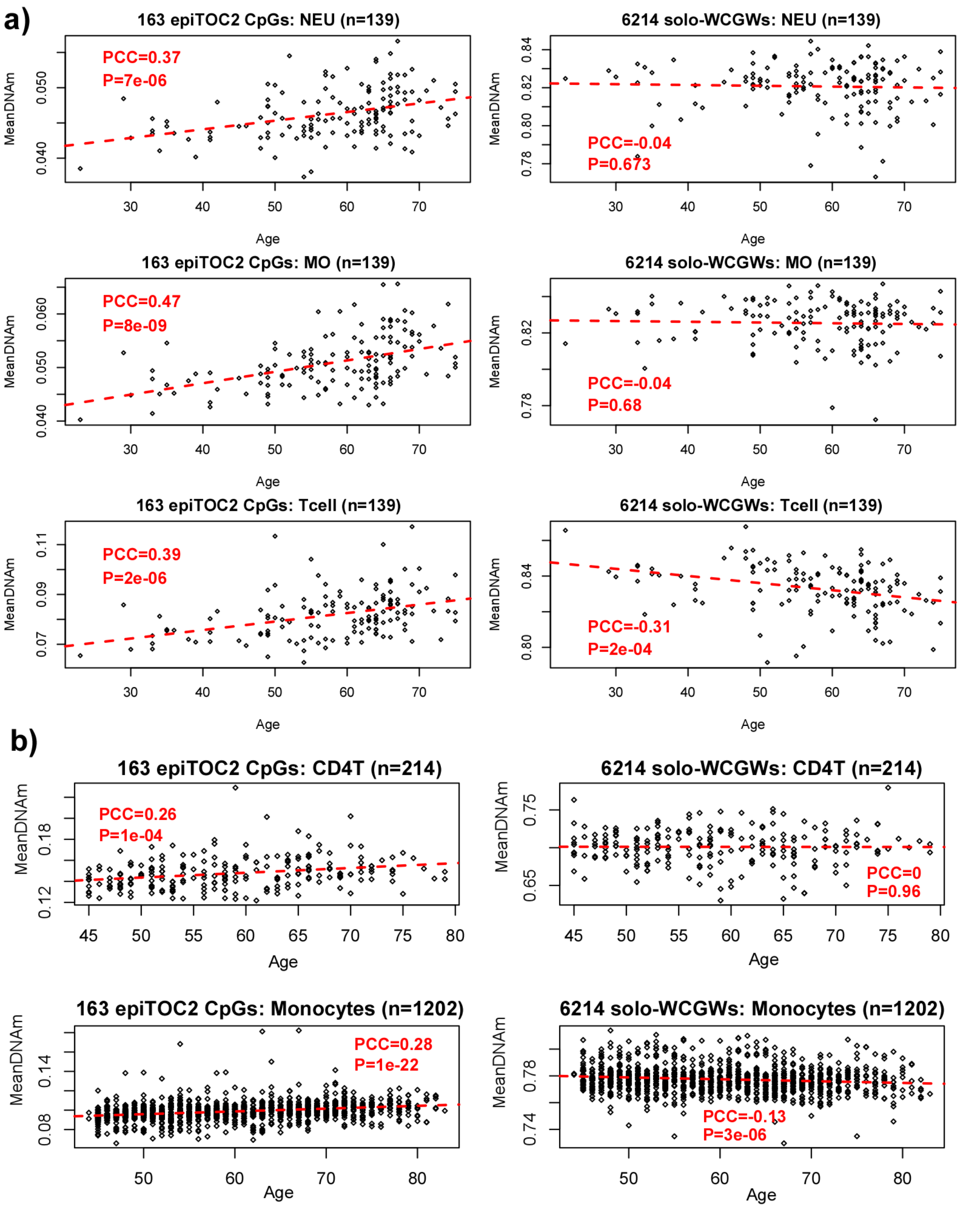

fig.S13: Associations of average DNAm over epiTOC2 and PMD solo-WCGWs with age in purified cell-types. a) Scatterplots of average DNAm (y-axis) over epiTOC2 or PMD solo-WCGWs against chronological age in the Blueprint Illumina 450k DNAm dataset [8]. Pearson Correlation Coefficient (PCC) and P-value from a linear regression are given. **b)** As a), but for the Reynolds Illumina 450k DNAm dataset [3] .

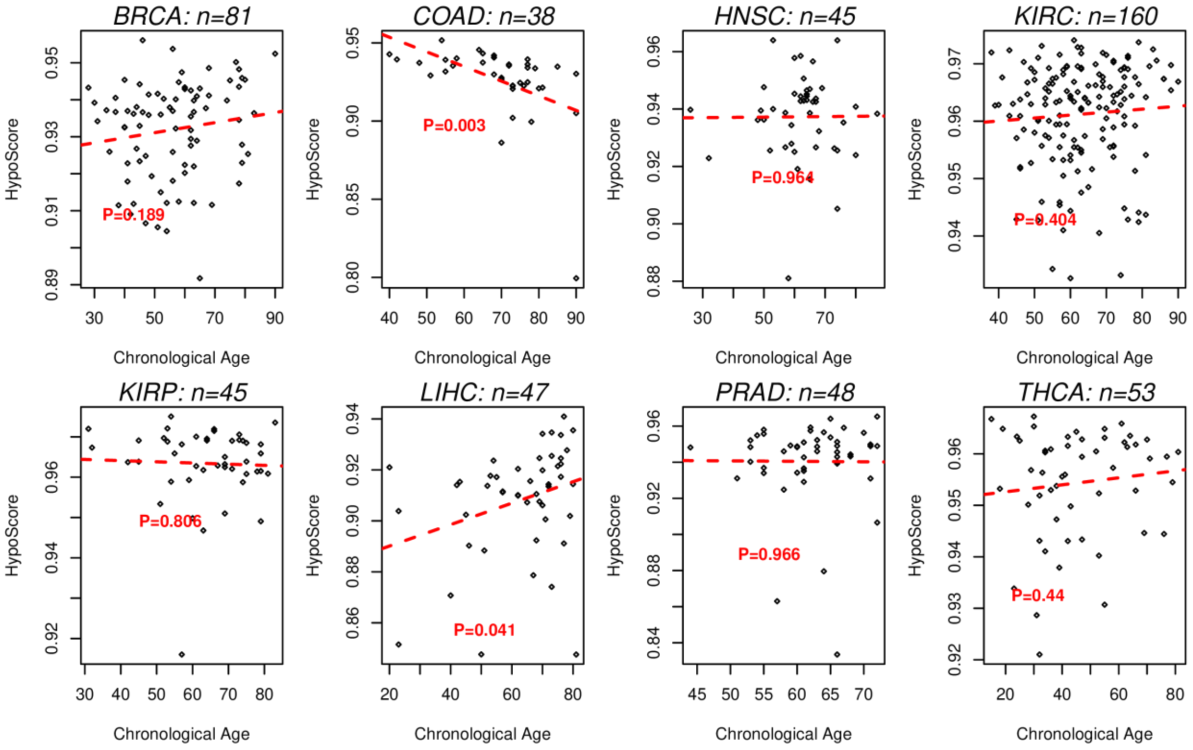

fig.S14: No consistent anti-correlation between HypoClock score and chronological age in normal-adjacent tissue from TCGA. Scatterplots of the HypoScore (i.e. average DNAm over the 678 solo-WCGWs) [y-axis] versus chronological age [x-axis] for normal-adjacent tissues from 8 TCGA cancer-types for which reasonable numbers of normal-adjacent samples were available. Number of samples given above plot. Estimated regression line and P-value are given in red.

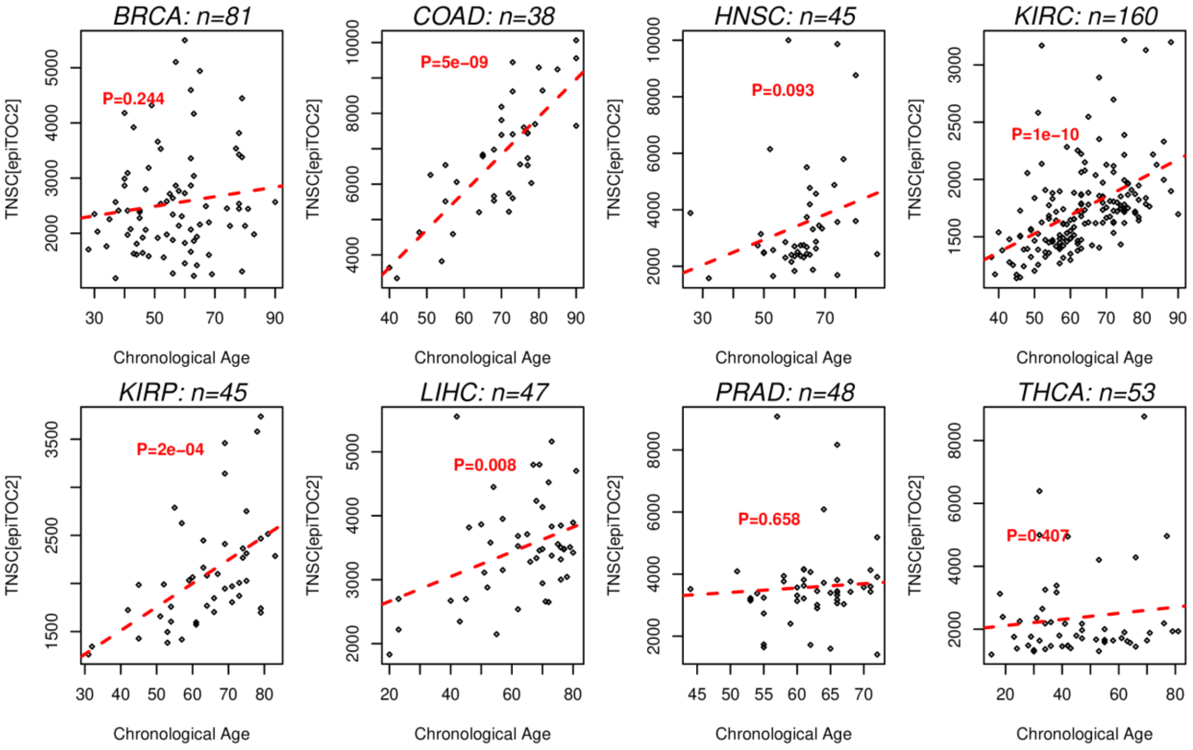

fig.S15: Correlation of epiTOC2 scores with chronological age in normal-adjacent tissue from TCGA. Scatterplots of the total number of stem-cell divisions (TNSC) as estimated using epiTOC2 [y-axis] versus chronological age [x-axis] for normal-adjacent tissues from 8 TCGA cancer-types for which reasonable numbers of normal-adjacent samples were available. Number of samples given above plot. Estimated regression line and P-value are given in red.

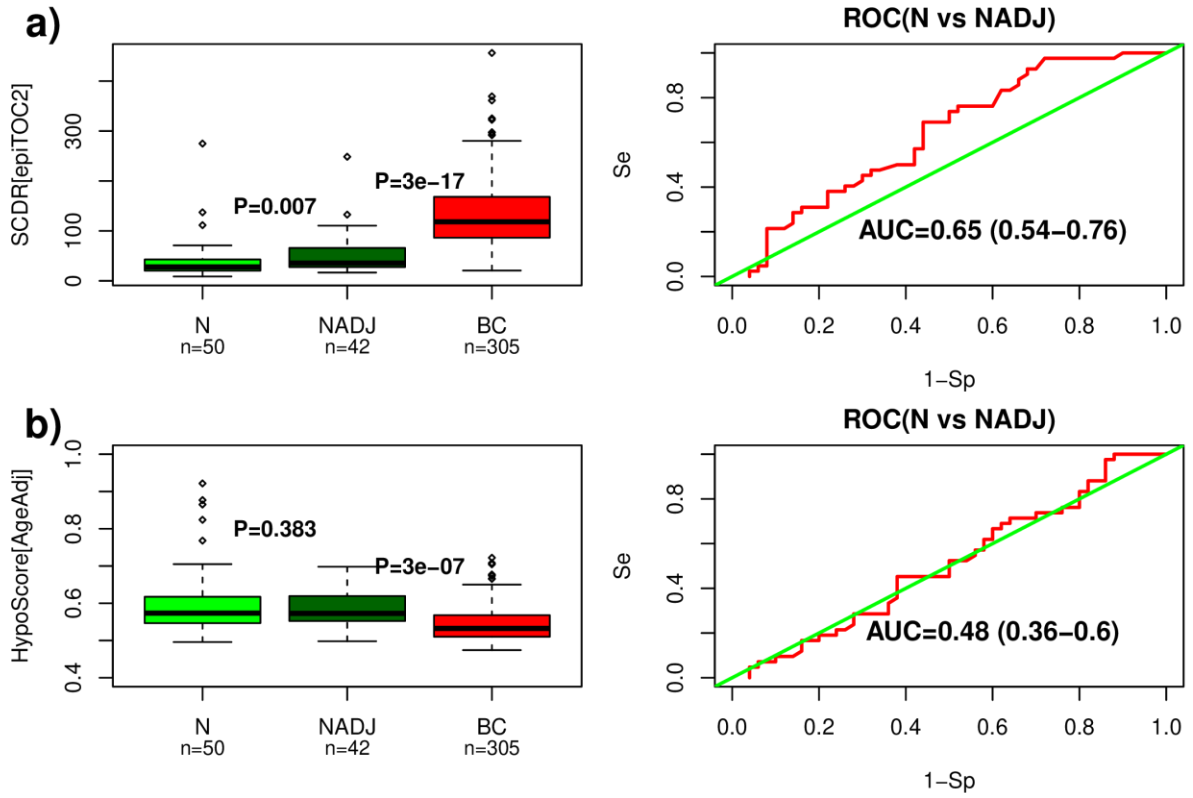

fig.S16: Comparison between epiTOC2 and HypoClock in breast tissue. a) Left panel: Estimated stem-cell division rate (SCDR) from epiTOC2 (y-axis) against disease status of breast tissue (x-axis, N=normal breast from healthy woman, NADJ=normal breast adjacent to cancer, BC=breast cancer). The number of samples in each group is given. P-values derive from one-tailed Wilcoxon-rank sum tests comparing neighboring progression stages. **Right panel:** ROC curve, AUC value and 95% confidence interval for SCDR discriminating normal from NADJ. **b)** As a), but for the HypoClock model.

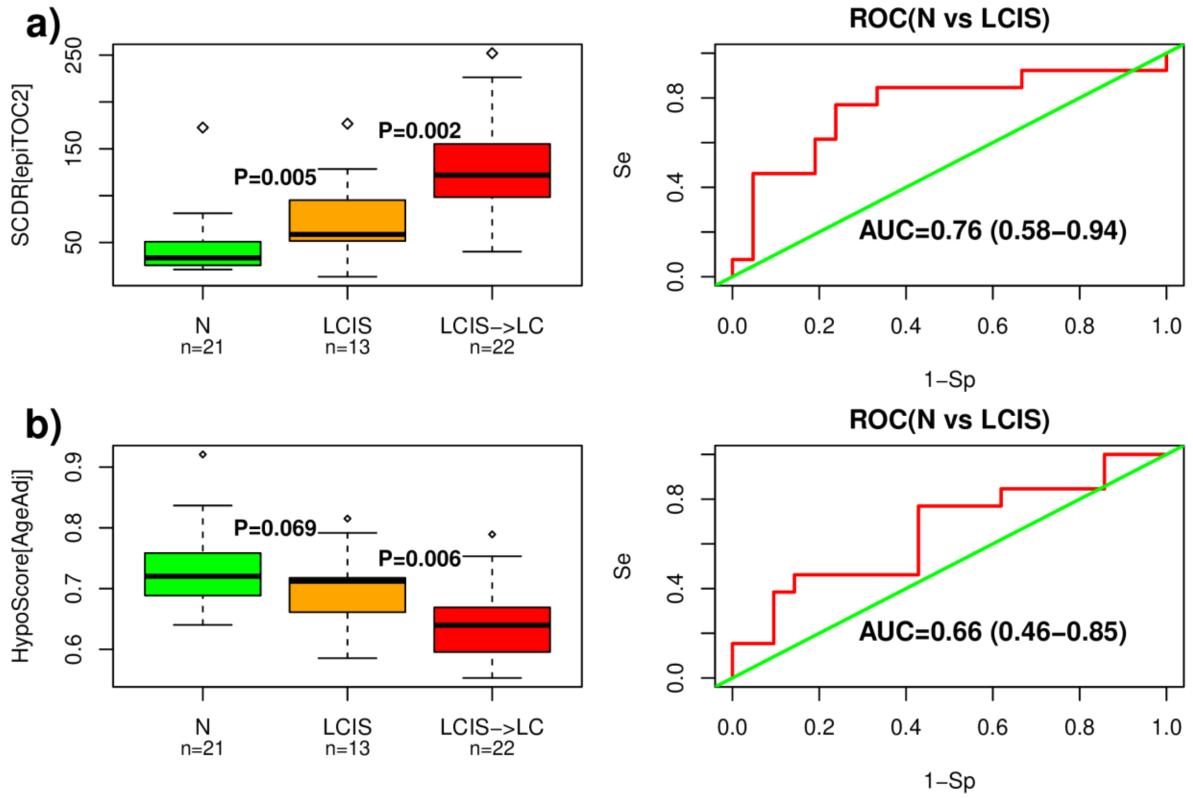

fig.S17: Comparison between epiTOC2 and HypoClock in lung tissue. a) Left panel: Estimated stem-cell division rate (SCDR) from epiTOC2 (y-axis) against disease status of lung tissue (x-axis, N=normal lung from healthy woman, LCIS=lung carcinoma in situ which did not progress, LCIS->LC=lung carcinoma in situ which did progress to invasive lung cancer). The number of samples in each group is given. P-values derive from one-tailed Wilcoxon-rank sum tests comparing neighboring progression stages. **Right panel:** ROC curve, AUC value and 95% confidence interval for SCDR discriminating normal from LCIS. **b)** As a), but for the HypoClock model.

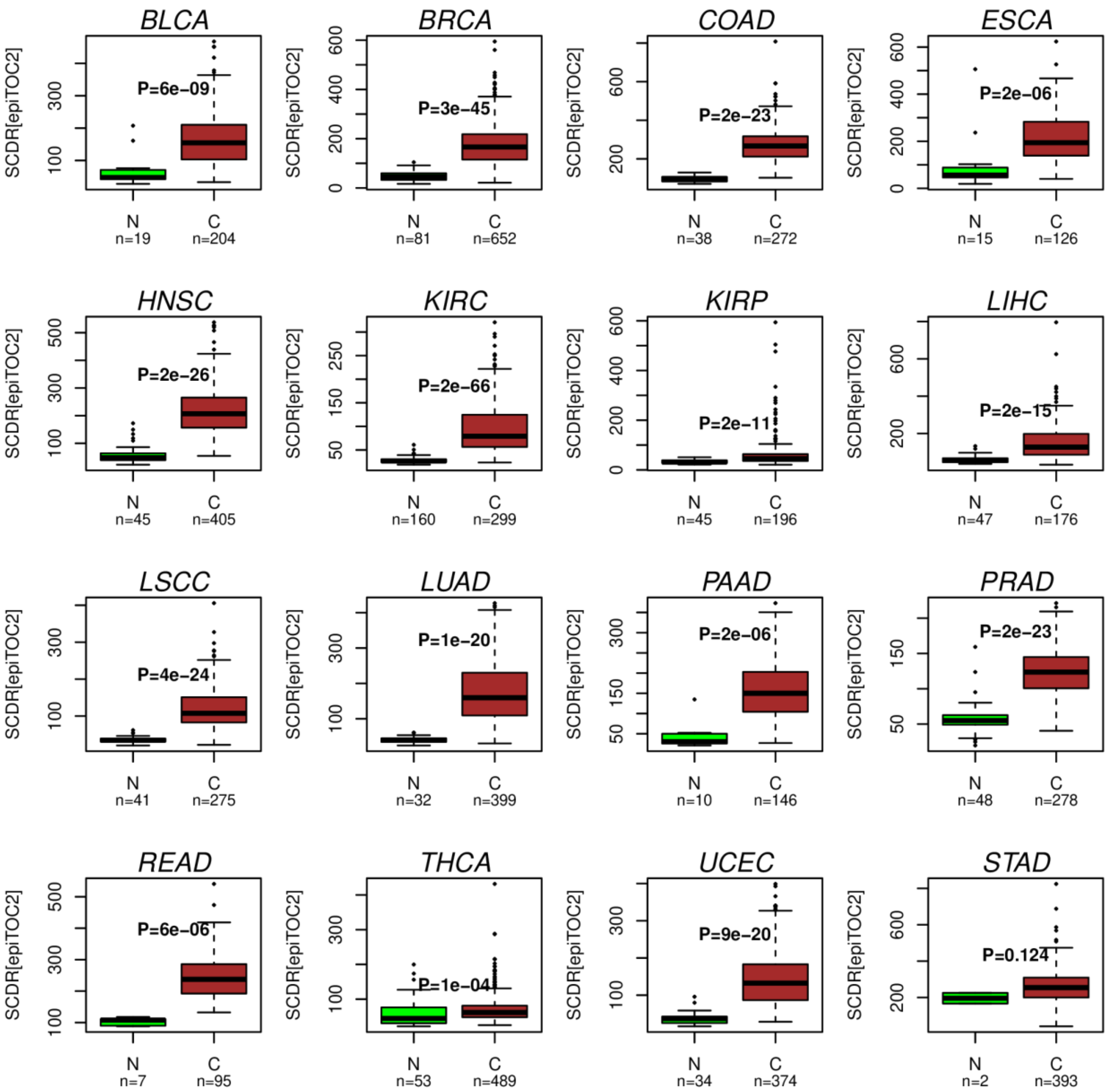

fig.S18: epiTOC2 predicts increased mitotic rate in cancer. For 16 TCGA cancer-types, we compare the estimated stem-cell division rate (SCDR) per stem-cell per year [y-axis] in each of the normal tissue samples (N) and corresponding cancers (C). By definition and construction, the SCDR is independent of chronological age. P-value from a one-tailed Wilcoxon test is given. Number of normal and cancer samples are given.

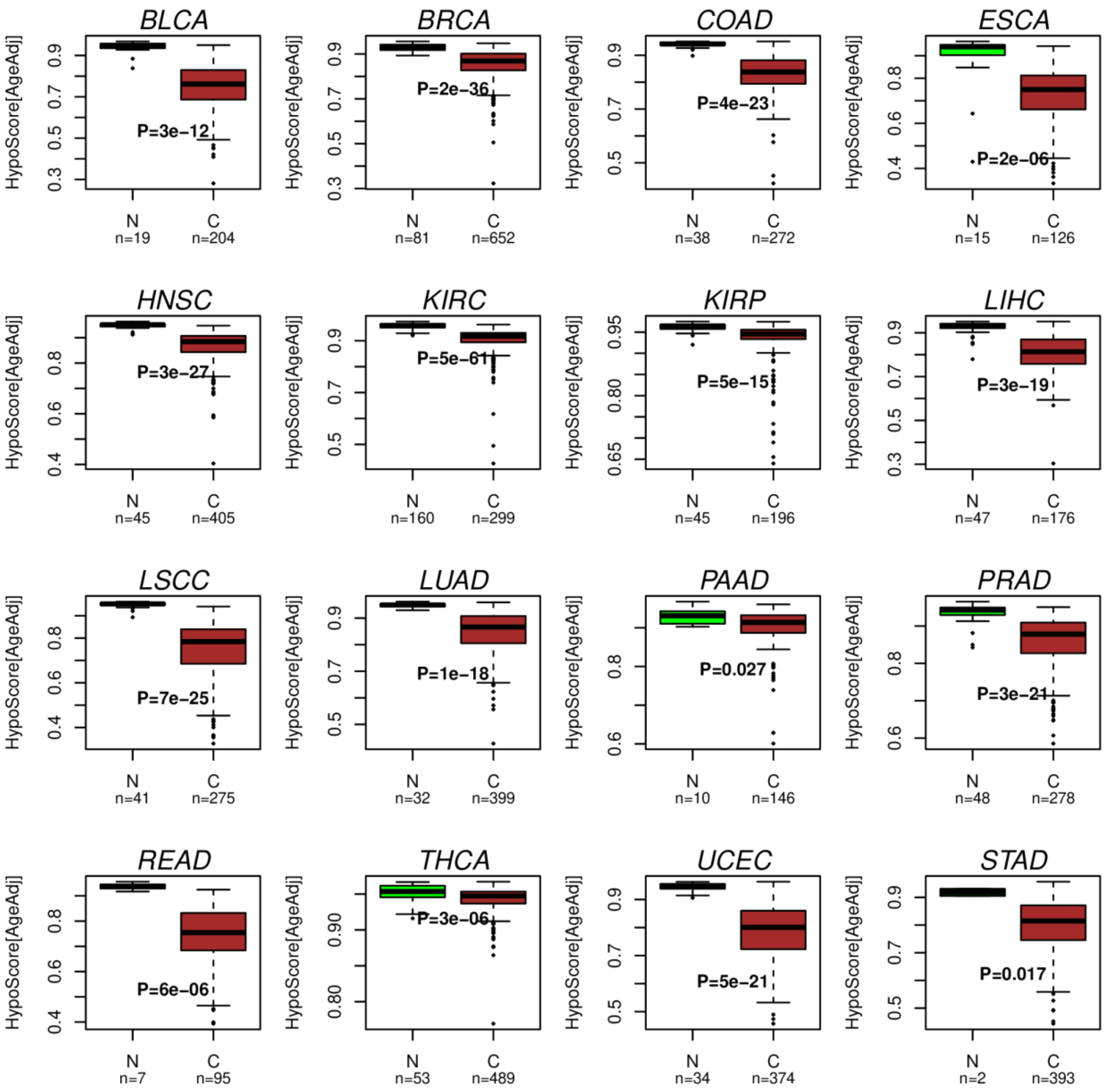

fig.S19: Associations of HypoClock-score with normal/cancer status in samples from TCGA. For 16 TCGA cancer-types, we compare the age-adjusted HypoClock score [HypoScore, y-axis] in each of the normal tissue samples (N) and corresponding cancers (C). By definition and construction, the HypoScore is independent of chronological age. P-value from a one-tailed Wilcoxon test is given. Number of normal and cancer samples are given.

SUPPLEMENTARY TABLES

epiTOC2-CpG	delta	IR	delta*IR	beta0	Gene Name	Relation
cg00043095	0.001	2	0.002	0.01	SGPP2	Island
cg00347369	5.00E-05	40	0.002	0.05	STMN2	N_Shore
cg00397986	5.00E-05	45	0.00225	0.05	GATA4	Island
cg00466268	0.00075	5	0.00375	0.05	SLITRK3	
cg00884606	5.00E-05	45	0.00225	0.05	NOL4	Island
cg00916884	0.001	2	0.002	0.05	MT1M	Island
cg01435574	5.00E-05	40	0.002	0.05	BARX2	Island
cg01537995	5.00E-05	45	0.00225	0.04	FLRT2	N_Shore
cg01587896	5.00E-05	55	0.00275	0.05	ISL1	Island
cg01699217	5.00E-05	35	0.00175	0.05	BHLHE23	Island
cg01783070	0.00025	5	0.00125	0.05	PAX1	Island
cg01830294	1.00E-04	60	0.006	0.05	WNT2;WNT2	Island
cg02150988	5.00E-05	55	0.00275	0.05	HOXD1	Island
cg02186542	5.00E-05	40	0.002	0.05	ADAMTS18	S_Shore
cg02266732	0.001	1	0.001	0.04	HTR1A	Island
cg02631468	5.00E-05	40	0.002	0.05	VSX1;VSX1	Island
cg02726121	1.00E-05	100	0.001	0.05	GATA4	Island
cg02796545	5.00E-04	5	0.0025	0.05	KL	Island
cg02964724	5.00E-05	35	0.00175	0.05	ASCL2	Island
cg03045635	0.00075	10	0.0075	0.05	DRD5	Island
cg03111498	5.00E-05	60	0.003	0.05	VSX1;VSX1	Island
cg03140968	0.00025	5	0.00125	0.05	PAX1	Island
cg03181582	5.00E-05	35	0.00175	0.05	ABCC8	Island
cg03430846	5.00E-05	35	0.00175	0.05	NRG1	Island
cg03450948	5.00E-05	55	0.00275	0.05	HOXD1	Island
cg03603951	0.001	1	0.001	0.03	KCNK12	Island
cg03874199	5.00E-05	35	0.00175	0.03	HOXD12	Island
cg04188273	1.00E-05	100	0.001	0	COL25A1;COL25A1	Island
cg04408488	0.001	1	0.001	0.04	PDX1	Island
cg04431946	0.001	2	0.002	0.03	KCNK12	Island
cg04549460	0.001	1	0.001	0.01	RSPO2	S_Shore
cg04597433	5.00E-05	85	0.00425	0.05	DRD5	Island
cg04633225	0.001	1	0.001	0.05	PDX1	Island
cg04672706	5.00E-05	30	0.0015	0.02	SOX17	Island
cg04902729	5.00E-05	35	0.00175	0.03	CACNA1E	Island
cg04996219	5.00E-05	25	0.00125	0	CTNND2	Island
cg05093169	5.00E-05	65	0.00325	0.05	PHOX2A	Island
cg05099387	5.00E-05	75	0.00375	0.05	HOXD1	Island
cg05182249	5.00E-05	35	0.00175	0.04	KCNK13	Island
cg05344430	5.00E-05	30	0.0015	0	GSX1	Island

cg05446424	0.001	1	0.001	0.04	FAM84A	Island
cg05666607	0.001	1	0.001	0.02	POU4F3	Island
cg05886671	0.00075	2	0.0015	0.05	NEUROG3	Island
cg06469345	5.00E-05	65	0.00325	0.05	DRD5	Island
cg06672560	0.00075	3	0.00225	0.05	CBLN4	Island
cg06987468	5.00E-05	95	0.00475	0.05	WNT2;WNT2	Island
cg07195011	5.00E-05	30	0.0015	0	CTNND2	Island
cg07315745	5.00E-05	45	0.00225	0.05	BARX2	Island
cg07357987	0.001	2	0.002	0.05	SGPP2	Island
cg07557260	5.00E-05	55	0.00275	0.05	ADCY8	Island
cg07621749	0.001	2	0.002	0.05	LTK;LTK;LTK	S_Shore
cg07897248	0.00025	5	0.00125	0.05	DCC	N_Shore
cg07950000	0.00075	5	0.00375	0.05	GRIK1;GRIK1	S_Shore
cg08073312	5.00E-05	25	0.00125	0.04	SOX14	Island
cg08074851	5.00E-05	45	0.00225	0.05	KCNK12	Island
cg08448701	0.00075	2	0.0015	0.05	PAX1	Island
cg08530317	5.00E-05	35	0.00175	0.04	SLC6A5	N_Shore
cg08834401	0.001	2	0.002	0.05	FIGLA	Island
cg08961408	5.00E-05	25	0.00125	0.02	NPNT	Island
cg09578028	5.00E-05	30	0.0015	0.05	HOXD9	Island
cg10094616	5.00E-05	30	0.0015	0.03	FAM150A	Island
cg10281002	5.00E-05	35	0.00175	0.05	TBX5;TBX5	Island
cg10343742	0.00025	25	0.00625	0.05	SOX14	Island
cg10406295	0.00025	5	0.00125	0.03	SFRP1	Island
cg10824063	5.00E-05	35	0.00175	0.05	NELL1;NELL1	Island
cg11334771	0.001	1	0.001	0.05	PHOX2A	Island
cg11354629	5.00E-05	30	0.0015	0.05	GSX1	Island
cg11723848	5.00E-05	35	0.00175	0.01	UNC5C	Island
cg11848563	5.00E-05	35	0.00175	0.05	FOXD3	Island
cg12071328	5.00E-05	55	0.00275	0.05	NELL1;NELL1	Island
cg12180703	5.00E-05	60	0.003	0.05	SLC32A1	Island
cg12417685	1.00E-05	100	0.001	0	FAM19A4;FAM19A4	Island
cg12743978	5.00E-05	85	0.00425	0.05	CHODL	Island
cg12882697	5.00E-05	35	0.00175	0.03	SLC6A3	Island
cg13078140	5.00E-04	5	0.0025	0.05	SLIT2	Island
cg13177747	0.00025	5	0.00125	0.05	RSPO2	S_Shore
cg13281139	0.00075	2	0.0015	0.05	SLIT2	Island
cg13368756	5.00E-05	40	0.002	0	CTNND2	Island
cg13526007	5.00E-05	35	0.00175	0.05	LRFN5	S_Shore
cg14456683	1.00E-04	95	0.0095	0.05	ZIC1	Island
cg14473102	5.00E-05	55	0.00275	0.05	HOXD8	Island
cg14717170	0.00025	5	0.00125	0.04	SLITRK3	
cg14769207	5.00E-05	70	0.0035	0.05	SIM2;SIM2	Island
cg14834938	5.00E-05	50	0.0025	0.05	ISL1	Island

cg14991487	1.00E-05	100	0.001	0.01	HOXD9	Island
cg15119027	5.00E-05	55	0.00275	0.05	FGF3	Island
cg15186181	0.001	3	0.003	0.05	SOX17	Island
cg15237923	0.00025	5	0.00125	0.05	CSMD1	Island
cg15272362	5.00E-05	35	0.00175	0.05	VSX1;VSX1	Island
cg15493780	0.001	2	0.002	0.05	PRLHR	Island
cg15984718	5.00E-05	35	0.00175	0.05	UNC5C	Island
cg16023545	5.00E-05	70	0.0035	0.05	GBX2	Island
cg16033053	5.00E-05	30	0.0015	0.05	MKX	Island
cg16523380	5.00E-05	40	0.002	0.05	CASZ1;CASZ1	Island
cg16800165	5.00E-05	35	0.00175	0.01	FLRT2	N_Shore
cg17037282	5.00E-05	90	0.0045	0.05	ISL1	Island
cg17076890	1.00E-04	60	0.006	0.05	PTF1A	Island
cg17152757	5.00E-05	35	0.00175	0.04	GHSR;GHSR	Island
cg17339147	0.001	2	0.002	0.05	WNT2;WNT2	Island
cg17371081	5.00E-05	35	0.00175	0.05	NELL1;NELL1	Island
cg17412886	5.00E-05	55	0.00275	0.05	ISL1	Island
cg17816908	5.00E-05	40	0.002	0.05	SFRP1	Island
cg17863912	5.00E-05	25	0.00125	0.04	HOXD8	Island
cg18097532	1.00E-04	35	0.0035	0.05	ABCC8	Island
cg18369866	0.001	1	0.001	0.03	POU4F3	Island
cg18498593	1.00E-05	100	0.001	0.04	HOXB13	Island
cg19042459	0.00025	5	0.00125	0.05	DCC	N_Shelf
cg19054524	0.001	1	0.001	0.04	PAX1	Island
cg19180624	5.00E-05	35	0.00175	0.02	HOXD1	Island
cg19283196	5.00E-05	70	0.0035	0.05	SLC10A4	N_Shore
cg19346645	5.00E-05	60	0.003	0.05	UCP1	Island
cg19384289	0.00025	5	0.00125	0.05	HOXD8	Island
cg19401340	0.00025	5	0.00125	0.01	PPM1E	Island
cg19542816	5.00E-05	40	0.002	0.04	HOXD1	Island
cg19711579	5.00E-05	40	0.002	0	NEUROD1	N_Shelf
cg19712603	0.001	1	0.001	0.03	PCDH8;PCDH8	Island
cg19761848	0.001	4	0.004	0.05	GBX2	Island
cg20097440	5.00E-05	35	0.00175	0.05	CYP26B1	Island
cg20585530	0.001	2	0.002	0.05	SIX6	Island
cg20720059	0.00025	5	0.00125	0.04	FAM84A	Island
cg20926035	5.00E-05	35	0.00175	0.05	SIX2	Island
cg21053529	0.00075	3	0.00225	0.05	GJD2	Island
cg21269843	5.00E-05	30	0.0015	0.05	OTP	Island
cg21426003	0.00075	2	0.0015	0.05	GBX2	Island
cg21517947	5.00E-05	30	0.0015	0.01	SFRP1	Island
cg21859781	5.00E-05	30	0.0015	0.02	GDF6	Island
cg22240472	0.001	1	0.001	0.05	ABCC8	Island
cg22274395	0.001	1	0.001	0.01	UCP1	Island

cg22277994	5.00E-05	35	0.00175	0.05	SSTR2	Island
cg22428147	5.00E-05	30	0.0015	0.05	DGKI	Island
cg22600043	5.00E-05	100	0.005	0.05	RSPO2	S_Shore
cg22653976	1.00E-05	100	0.001	0.02	MYOD1	Island
cg22797735	0.00075	2	0.0015	0.01	TBX3;TBX3	Island
cg23132624	5.00E-05	30	0.0015	0.04	KL	Island
cg23217126	0.001	1	0.001	0.03	DOK6	Island
cg23335460	5.00E-05	55	0.00275	0.05	CRTAC1	Island
cg23405575	0.00075	2	0.0015	0.01	SLC6A1	N_Shore
cg23420260	0.00075	3	0.00225	0.05	HOXD1	Island
cg23774356	5.00E-05	35	0.00175	0.05	GSC2	Island
cg23847712	1.00E-04	65	0.0065	0.05	DRD5	Island
cg24154839	0.001	2	0.002	0.05	GABRA4	Island
cg24319902	5.00E-05	55	0.00275	0.05	SFRP1	Island
cg24891539	0.00075	2	0.0015	0.05	SOX17	Island
cg24989962	5.00E-04	5	0.0025	0.05	PTGDR	Island
cg25116388	5.00E-05	45	0.00225	0.05	HOXB8	S_Shore
cg25307168	0.00075	2	0.0015	0.05	SLC32A1	Island
cg25599538	1.00E-05	100	0.001	0.01	UCP1	Island
cg25640822	5.00E-05	45	0.00225	0.05	NEUROG1	Island
cg25682299	0.001	1	0.001	0.05	POU4F1	Island
cg25951981	5.00E-05	25	0.00125	0.04	GABRA4	Island
cg25993718	0.001	3	0.003	0.05	CBLN4	Island
cg26124980	5.00E-05	25	0.00125	0.04	ATOH1	Island
cg26332560	5.00E-05	45	0.00225	0.05	ADCY8	Island
cg26410450	5.00E-05	45	0.00225	0.05	MYOD1	Island
cg26554592	5.00E-04	5	0.0025	0.05	SORCS1;SORCS1	Island
cg26565021	0.00075	2	0.0015	0.05	CHST8;CHST8	Island
cg27058486	5.00E-05	45	0.00225	0.05	CHST8;CHST8	Island
cg27176138	5.00E-05	60	0.003	0.05	HS6ST3	Island
cg27254482	0.00075	3	0.00225	0.05	OLIG2	Island
cg27363829	5.00E-05	60	0.003	0.05	SLITRK3	
cg27505273	5.00E-05	30	0.0015	0.04	SOX14	Island
cg27625055	1.00E-05	100	0.001	0.02	PTPRT;PTPRT	Island
cg27648075	5.00E-05	40	0.002	0.05	DSC3;DSC3	Island

table.S1: Estimated epiTOC2 parameters. Table lists the estimated probabilities of de-novo methylation (*delta*), the estimated intrinsic rate (*IR*) of stem-cell division in blood (i.e number of stem-cell divisions per stem-cell per year), the product of *delta*IR,* and the ground-state methylation (*beta0*), i.e. the estimated methylation at fetal stage, for a total of 163 CpGs, which map to PRC2 targets and which are constitutively unmethylated across 11 fetal tissue types (beta < 0.2 for all fetal tissues including cord-blood), and for which the product *delta*IR* ≥ *0.001.*

CpG	delta	beta0	IR	deltaBeta(Age=80)
cg00043095	5.00E-05	0.02	35	0.066
cg00347369	5.00E-05	0.05	35	0.064
cg00397986	5.00E-05	0.05	35	0.064
cg00466268	1.00E-04	0.05	35	0.124
cg00884606	1.00E-04	0.01	35	0.129
cg00916884	5.00E-05	0.05	35	0.064
cg01435574	5.00E-05	0.05	35	0.064
cg01537995	5.00E-05	0.05	35	0.064
cg01587896	1.00E-04	0.03	35	0.127
cg01699217	5.00E-05	0.05	35	0.064
cg01783070	5.00E-05	0.04	35	0.065
cg01830294	0.00025	0	35	0.295
cg02150988	1.00E-04	0.03	35	0.127
cg02186542	5.00E-05	0.05	35	0.064
cg02266732	5.00E-05	0.02	35	0.066
cg02631468	5.00E-05	0.05	35	0.064
cg02726121	5.00E-05	0.03	35	0.066
cg02796545	1.00E-04	0.02	35	0.128
cg02964724	5.00E-05	0.05	35	0.064
cg03045635	0.00025	0.03	35	0.286
cg03111498	1.00E-04	0.03	35	0.127
cg03140968	5.00E-05	0.04	35	0.065
cg03181582	5.00E-05	0.05	35	0.064
cg03430846	5.00E-05	0.05	35	0.064
cg03450948	1.00E-04	0.03	35	0.127
cg03603951	5.00E-05	0.01	35	0.067
cg03874199	5.00E-05	0.03	35	0.066
cg04188273	1.00E-05	0.02	35	0.014
cg04408488	5.00E-05	0.02	35	0.066
cg04431946	5.00E-05	0.04	35	0.065
cg04549460	1.00E-05	0.03	35	0.013
cg04597433	1.00E-04	0.05	35	0.124
cg04633225	5.00E-05	0.03	35	0.066
cg04672706	5.00E-05	0.01	35	0.067
cg04902729	5.00E-05	0.03	35	0.066

cg04996219	1.00E-05	0.03	35	0.013
cg05093169	1.00E-04	0.05	35	0.124
cg05099387	1.00E-04	0.05	35	0.124
cg05182249	5.00E-05	0.04	35	0.065
cg05344430	1.00E-05	0.04	35	0.013
cg05446424	5.00E-05	0.02	35	0.066
cg05666607	5.00E-05	0	35	0.068
cg05886671	5.00E-05	0.05	35	0.064
cg06469345	1.00E-04	0.04	35	0.125
cg06672560	5.00E-05	0.05	35	0.064
cg06987468	1.00E-04	0.05	35	0.124
cg07195011	1.00E-05	0.03	35	0.013
cg07315745	1.00E-04	0.01	35	0.129
cg07357987	5.00E-05	0.05	35	0.064
cg07557260	1.00E-04	0.03	35	0.127
cg07621749	5.00E-05	0.05	35	0.064
cg07897248	5.00E-05	0.04	35	0.065
cg07950000	1.00E-04	0.05	35	0.124
cg08073312	5.00E-05	0.02	35	0.066
cg08074851	1.00E-04	0.01	35	0.129
cg08448701	5.00E-05	0.05	35	0.064
cg08530317	5.00E-05	0.04	35	0.065
cg08834401	5.00E-05	0.05	35	0.064
cg08961408	5.00E-05	0	35	0.068
cg09578028	5.00E-05	0.04	35	0.065
cg10094616	5.00E-05	0.02	35	0.066
cg10281002	5.00E-05	0.05	35	0.064
cg10343742	0.00025	0	35	0.295
cg10406295	5.00E-05	0.02	35	0.066
cg10824063	5.00E-05	0.05	35	0.064
cg11334771	5.00E-05	0.03	35	0.066
cg11354629	5.00E-05	0.04	35	0.065
cg11723848	5.00E-05	0.01	35	0.067
cg11848563	5.00E-05	0.05	35	0.064
cg12071328	1.00E-04	0.03	35	0.127
cg12180703	1.00E-04	0.04	35	0.125
cg12417685	1.00E-05	0.02	35	0.014

cg12743978	1.00E-04	0.05	35	0.124
cg12882697	5.00E-05	0.03	35	0.066
cg13078140	1.00E-04	0.02	35	0.128
cg13177747	5.00E-05	0.04	35	0.065
cg13281139	5.00E-05	0.04	35	0.065
cg13368756	5.00E-05	0.01	35	0.067
cg13526007	5.00E-05	0.05	35	0.064
cg14456683	0.00025	0.05	35	0.281
cg14473102	1.00E-04	0.03	35	0.127
cg14717170	5.00E-05	0.03	35	0.066
cg14769207	1.00E-04	0.05	35	0.124
cg14834938	1.00E-04	0.02	35	0.128
cg14991487	1.00E-05	0.03	35	0.013
cg15119027	1.00E-04	0.03	35	0.127
cg15186181	1.00E-04	0.04	35	0.125
cg15237923	5.00E-05	0.04	35	0.065
cg15272362	5.00E-05	0.05	35	0.064
cg15493780	5.00E-05	0.05	35	0.064
cg15984718	5.00E-05	0.05	35	0.064
cg16023545	1.00E-04	0.05	35	0.124
cg16033053	5.00E-05	0.04	35	0.065
cg16523380	5.00E-05	0.05	35	0.064
cg16800165	5.00E-05	0.01	35	0.067
cg17037282	1.00E-04	0.05	35	0.124
cg17076890	0.00025	0	35	0.295
cg17152757	5.00E-05	0.04	35	0.065
cg17339147	5.00E-05	0.05	35	0.064
cg17371081	5.00E-05	0.05	35	0.064
cg17412886	1.00E-04	0.03	35	0.127
cg17816908	5.00E-05	0.05	35	0.064
cg17863912	5.00E-05	0.02	35	0.066
cg18097532	1.00E-04	0.05	35	0.124
cg18369866	5.00E-05	0.01	35	0.067
cg18498593	5.00E-05	0.02	35	0.066
cg19042459	5.00E-05	0.04	35	0.065
cg19054524	5.00E-05	0.02	35	0.066
cg19180624	5.00E-05	0.02	35	0.066

cg19283196	1.00E-04	0.05	35	0.124
cg19346645	1.00E-04	0.03	35	0.127
cg19384289	5.00E-05	0.04	35	0.065
cg19401340	5.00E-05	0	35	0.068
cg19542816	5.00E-05	0.05	35	0.064
cg19711579	5.00E-05	0.01	35	0.067
cg19712603	5.00E-05	0.01	35	0.067
cg19761848	1.00E-04	0.05	35	0.124
cg20097440	5.00E-05	0.05	35	0.064
cg20585530	1.00E-04	0.01	35	0.129
cg20720059	5.00E-05	0.03	35	0.066
cg20926035	5.00E-05	0.05	35	0.064
cg21053529	1.00E-04	0.01	35	0.129
cg21269843	5.00E-05	0.04	35	0.065
cg21426003	5.00E-05	0.04	35	0.065
cg21517947	5.00E-05	0	35	0.068
cg21859781	5.00E-05	0.01	35	0.067
cg22240472	5.00E-05	0.03	35	0.066
cg22274395	1.00E-05	0.03	35	0.013
cg22277994	5.00E-05	0.05	35	0.064
cg22428147	5.00E-05	0.04	35	0.065
cg22600043	1.00E-04	0.05	35	0.124
cg22653976	1.00E-05	0.04	35	0.013
cg22797735	5.00E-05	0	35	0.068
cg23132624	5.00E-05	0.03	35	0.066
cg23217126	5.00E-05	0.01	35	0.067
cg23335460	1.00E-04	0.03	35	0.127
cg23405575	5.00E-05	0	35	0.068
cg23420260	1.00E-04	0.01	35	0.129
cg23774356	5.00E-05	0.05	35	0.064
cg23847712	0.00025	0	35	0.295
cg24154839	5.00E-05	0.05	35	0.064
cg24319902	1.00E-04	0.03	35	0.127
cg24891539	5.00E-05	0.05	35	0.064
cg24989962	1.00E-04	0.03	35	0.127
cg25116388	5.00E-05	0.05	35	0.064
cg25307168	5.00E-05	0.05	35	0.064

cg25599538	1.00E-05	0.03	35	0.013
cg25640822	5.00E-05	0.05	35	0.064
cg25682299	5.00E-05	0.03	35	0.066
cg25951981	5.00E-05	0.02	35	0.066
cg25993718	1.00E-04	0.04	35	0.125
cg26124980	5.00E-05	0.02	35	0.066
cg26332560	5.00E-05	0.05	35	0.064
cg26410450	5.00E-05	0.05	35	0.064
cg26554592	1.00E-04	0.02	35	0.128
cg26565021	5.00E-05	0.04	35	0.065
cg27058486	1.00E-04	0.01	35	0.129
cg27176138	1.00E-04	0.03	35	0.127
cg27254482	1.00E-04	0.02	35	0.128
cg27363829	1.00E-04	0.04	35	0.125
cg27505273	5.00E-05	0.03	35	0.066
cg27625055	1.00E-05	0.04	35	0.013
cg27648075	5.00E-05	0.05	35	0.064

table.S2: Final epiTOC2 parameters. Table lists the final probabilities of de-novo methylation (*delta*) and ground-state methylation (*beta0*) for the 163 epiTOC2-CpGs. These final parameters were re-estimated by fixing the IR=35 for all CpGs, as required, since the intrinsic rate of stem-cell division should not depend on the CpG-site. The last column gives an estimate of the expected average difference in DNAm between an 80-year old person and a newborn.

Study-Name	Tissue	N	N-ADJ	Risk/ Precancer	C	Reference
Erlangen	Breast	50	42	0	305	Teschendorff et al Nat Commun.2016
MRC-NSHD	Whole Blood	152	0	0	0	Teschendorff et al JAMA Oncol.2015
MRC-NSHD	Buccal	790	0	0	0	Teschendorff et al JAMA Oncol.2015
Lung	Lung	21	0	35 (LCIS)	0	Teschendorff et al JAMA Oncol.2015
Liver	Liver	26	0	0	0	Horvath et al PNAS 2014
Skin	Skin	19	0	0	0	Vandiver et al Genome Biol.2015
NormalBreast	Breast	121	0	0	0	Song et al Oncotarget 2017
Esophagus	Esophagus	52	0	84(BE)	24	Luebeck et al Clin Epigenetics 2017
Liu	Whole Blood	335	0	0	0	Liu et al Nat Biotech.2013
Hannum	Whole Blood	656	0	0	0	Hannum et al Mol.Cell.2014
Colon	Colon	8	0	39(Adenoma)	0	Bormann et al Cell Rep.2018
Gastric	Stomach	61	0	130 (Metaplasia)	0	Huang et al Cancer Cell 2018

table.S3: Summary of normal-tissue (non-TCGA) collection. Table lists the Illumina 450k DNAm datasets profiling normal tissues, with the number of normal tissues indicated in column "N". We also give the number of other samples, which are either normal-adjacent (N-ADJ) to cancer, samples which are at relatively high risk of cancer development (Risk/Precancer) or cancer (C).

REFERENCES

1. Hannum, G. *et al.* Genome-wide methylation profiles reveal quantitative views of human aging rates. *Mol Cell* **49**, 359-67 (2013).
2. Liu, Y. *et al.* Epigenome-wide association data implicate DNA methylation as an intermediary of genetic risk in rheumatoid arthritis. *Nat Biotechnol* **31**, 142-7 (2013).
3. Reynolds, L.M. *et al.* Age-related variations in the methylome associated with gene expression in human monocytes and T cells. *Nat Commun* **5**, 5366 (2014).
4. Nazor, K.L. *et al.* Recurrent variations in DNA methylation in human pluripotent stem cells and their differentiated derivatives. *Cell Stem Cell* **10**, 620-34 (2012).
5. Yang, Z. *et al.* Correlation of an epigenetic mitotic clock with cancer risk. *Genome Biol* **17**, 205 (2016).
6. Zhou, W. *et al.* DNA methylation loss in late-replicating domains is linked to mitotic cell division. *Nat Genet* **50**, 591-602 (2018).
7. Farlik, M. *et al.* DNA Methylation Dynamics of Human Hematopoietic Stem Cell Differentiation. *Cell Stem*

Cell **19**, 808-822 (2016).

8. Chen, L. *et al.* Genetic Drivers of Epigenetic and Transcriptional Variation in Human Immune Cells. *Cell* **167**, 1398-1414 e24 (2016).

9. Tserel, L. *et al.* Age-related profiling of DNA methylation in CD8+ T cells reveals changes in immune response and transcriptional regulator genes. *Sci Rep* **5**, 13107 (2015).
